# Biosynthesis of angelyl-CoA in *Saccharomyces cerevisiae*

**DOI:** 10.1186/s12934-018-0925-8

**Published:** 2018-05-12

**Authors:** Roberta Callari, David Fischer, Harald Heider, Nora Weber

**Affiliations:** 1 Evolva SA, Duggingerstrasse 23, 4153 Reinach, Switzerland; 2 Department of Plant and Environmental Sciences, University of Copenhagen, 1871 Frederiksberg C, Denmark

**Keywords:** Angelyl-CoA, Angelate, Propionyl-CoA, Methylmalonyl-CoA, 2-Methylacetoacetyl-CoA, 3-Hydroxyl-2-methyl-butyryl-CoA, Metabolic engineering, CoA-ligase, *Euphorbia peplus*, Yeast

## Abstract

**Background:**

The angelic acid moiety represents an essential modification in many biologically active products. These products are commonly known as angelates and several studies have demonstrated their therapeutic benefits, including anti-inflammatory and anti-cancer effects. However, their availability for use in the development of therapeutics is limited due to poor extraction yields. Chemical synthesis has been achieved but its complexity prevents application, therefore microbial production may offer a promising alternative. Here, we engineered the budding yeast *Saccharomyces cerevisiae* to produce angelyl-CoA, the CoA-activated form of angelic acid.

**Results:**

For yeast-based production of angelyl-CoA we first expressed genes recently identified in the biosynthetic cluster *ssf* of *Streptomyces* sp. SF2575 in *S. cerevisiae*. Exogenous feeding of propionate and heterologous expression of a propionyl-CoA synthase from *Streptomyces* sp. were initially employed to increase the intracellular propionyl-CoA level, resulting in production of angelyl-CoA in the order of 5 mg/L. Substituting the *Streptomyces* sp. propionyl-CoA carboxylase with a carboxylase derived from *Streptomyces coelicolor* resulted in angelyl-CoA levels up to 6.4 mg/L. In vivo analysis allowed identification of important intermediates in the pathway, including methyl-malonyl-CoA and 3-hydroxyl-2-methyl-butyryl-CoA. Furthermore, methyl-malonate supplementation and expression of *matB* CoA ligase from *S. coelicolor* allowed for methyl-malonyl-CoA synthesis and supported, together with parts of the *ssf* pathway, angelyl-CoA titres of approximately 1.5 mg/L. Finally, feeding of angelic acid to yeasts expressing acyl-CoA ligases from plant species led to angelyl-CoA production rates of approximately 40 mg/L.

**Conclusions:**

Our results demonstrate the biosynthesis of angelyl-CoA in yeast from exogenously supplied carboxylic acid precursors. This is the first report on the activity of the *ssf* genes. We envision that our approach will provide a platform for a more sustainable production of the pharmaceutically important compound class of angelates.

**Electronic supplementary material:**

The online version of this article (10.1186/s12934-018-0925-8) contains supplementary material, which is available to authorized users.

## Background

Esters of angelic acid ((*Z*)-2-methyl-2-butenoic acid), also known as angelates, are pharmacologically active natural products widely distributed in plants (Additional file 1: Figure S1). The best-known and most studied example is certainly represented by ingenol-3-angelate (also known as ingenol-mebutate), a topical chemotherapeutic recently approved by the FDA for the treatment of actinic keratosis, a pre-cancerous skin condition. This ester of the diterpenoid ingenol and angelic acid is derived from the sap of *Euphorbia peplus*—the garden weed “petty spurge”, known as a traditional remedy for warts and basal cell carcinomas [1]. Other notable plant angelates include petasin and its isomers isopetasin and neopetasin, found in *Petasites hybridus*, a species used for years as herbal supplement for its anti-spasmodic and anti-inflammatory effects [2]. The cytotoxic gordonosides, angelic acid esters isolated from *Gordonia chrysandra,* are frequently applied in traditional medicine for treating diarrhea, gastralgia, and arthritis [3]. Decoursinol angelate, isolated from roots of the medicinal herb *Angelica gigas* Nakai, exerts anti-cancer and anti-inflammatory activities [4]. Thapsigargin, found in the roots of the mediterranean plant species *Thapsia garganica* L., is an inhibitor of the sarco-endoplasmic reticulum Ca^2+^-ATPase used in the treatment of solid tumors [5].

Recently, bacterial angelates have also been reported: SF2575 is a tetracycline polyketide-angelic acid ester produced by *Streptomyces* sp. SF2575 [6]. The compound exhibits not only weak antibiotic activity but also potent anti-cancer activity towards a broad range of cancer cell lines [7]. Trehangelins are trehalose angelates produced by the endophytic actinomycete *Polymorphospora rubra* K07-0510, displaying potent inhibitory activity against hemolysis of red blood cells [8].

Supply of angelates is currently based on extraction of the pure compounds from the species of origin. In some cases chemical synthesis has also been achieved [9–12]. However, both approaches are low yielding and have high environmental impact. In the case of ingenol-mebutate, direct isolation from the aerial tissue of *E. peplus* only yields 1.1 mg/kg of tissue [13]. Semi-synthesis, starting from the more abundant cognate compound ingenol, obtained an overall yield of around 31%, but relied on expensive catalysts [10]. Thapsigargin is isolated from wild plants of *T. garganica*, where it is present in minute amounts (1.2–1.5% of the dry weight, depending on the selected tissue) [5]. Its total synthesis in 42 steps from (*S*)-carvone had an overall yield of only 0.6% [9].

To make these compounds more accessible, microbial production certainly represents an interesting alternative route.

**Fig. 1:**
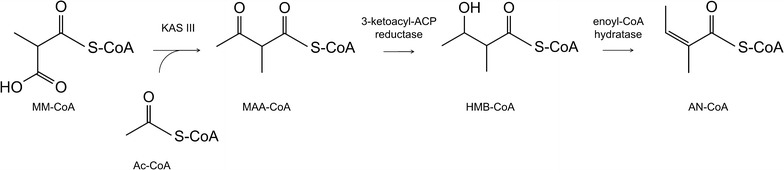
Bacterial biosynthetic pathway from MM-CoA to AN-CoA. MM-CoA—methylmalonyl-CoA; Ac-CoA—acetyl-CoA; MAA-CoA—2-methyl-acetoacetyl-CoA; HMB-CoA—3-hydroxyl-2-methyl-butyryl-CoA; AN-CoA—angelyl-CoA; KAS III—beta-ketoacyl-(acyl-carrier-protein) synthase III; 3-ketoacyl-ACP-reductase—3-ketoacyl-(acyl-carrier-protein) reductase

Unfortunately, biosynthesis of any of the plant angelates has not yet been elucidated and even the enzymes involved in the biosynthesis of the angelic acid moiety are unknown. In contrast, the gene clusters responsible for the bacterial synthesis of SF2575 (“*ssf*”) and trehangelin A (“*thg*”) have been identified and characterized [6, 14]. This has led to the elucidation of the metabolic pathways responsible for the biosynthesis of these compounds and the identification of enzymes needed for assembly of their core structures and also for tailoring reactions, including angelyl-CoA (AN-CoA) formation and esterification. The latter is synthesized in both *Streptomyces* sp. SF2575 and *P. rubra* by enzymes resembling those found in fatty acid biosynthesis. In joint action, the identified beta-ketoacyl-(acyl-carrier-protein) synthase III (KAS III), the 3-ketoacyl-(acyl-carrier-protein) reductase and the enoyl-CoA hydratase may lead to AN-CoA biosynthesis starting from acetyl-CoA (Ac-CoA) and methyl-malonyl-CoA (MM-CoA) via the intermediates 2-methyl-acetoacetyl-CoA (MAA-CoA) and 3-hydroxyl-2-methyl-butyryl-CoA (HMB-CoA) (Fig. 1). The enzymes from *P. rubra* were characterized in vitro [14], whereas the enzymes from *S.* sp. SF2575 have been suggested to be involved in AN-CoA formation based on homology to functionally similar enzymes from other species [6]. Pickens and co-workers [6] proposed that in *S.* sp. SF2575 condensation of MM-CoA and Ac-CoA to MMA-CoA is catalyzed by SsfN, a KAS III homolog. SsfK, homologous to 3-oxoacyl-ACP reductases, enables keto-reduction of MAA-CoA yielding HMB-CoA. In the last step, stereo-specific dehydration of HMB-CoA by SsfJ (a member of the enoyl-CoA hydratase/isomerase family) results in AN-CoA. Moreover, SsfE, homologous to biotin-dependent methyl-malonyl-CoA decarboxylases and propionyl-CoA carboxylases, was suggested to be involved in MM-CoA formation from Pr-CoA.

In this study we describe production of angelyl-CoA in the yeast *Saccharomyces cerevisiae.* This production host has well-established advantages when compared to other microorganisms such as robustness and resistance under harsh industrial conditions, resistance to phages and the ability to ferment sugars under acidic conditions. AN-CoA biosynthesis was achieved through expression of a heterologous pathway derived from the bacterial *ssf* cluster, allowing AN-CoA synthesis starting from propionyl-CoA. Upon engineering of the propionyl-CoA metabolism we reached maximum titres of approximately 6.4 mg/L AN-CoA. Moreover, the identification of acyl-CoA synthases from different plant sources enabled the use of angelic acid as substrate, yielding AN-CoA in titres of approximately 40 mg/L.

AN-CoA represents an important building block for the synthesis of many plant secondary metabolites with biological activity. AN-CoA is the substrate used by acyl-CoA transferases to catalyse esterification reactions, adding the angelate moiety onto diverse acceptor molecules. Its biosynthesis in yeast will enable angelyl-acylation of a broad range of compounds. Although further optimization is needed, we anticipate that the strains reported here will pave the way for the bio-based production of esters of angelic acid.

## Results

### Angelyl-CoA production in yeast starting from propionate

**Fig. 2:**
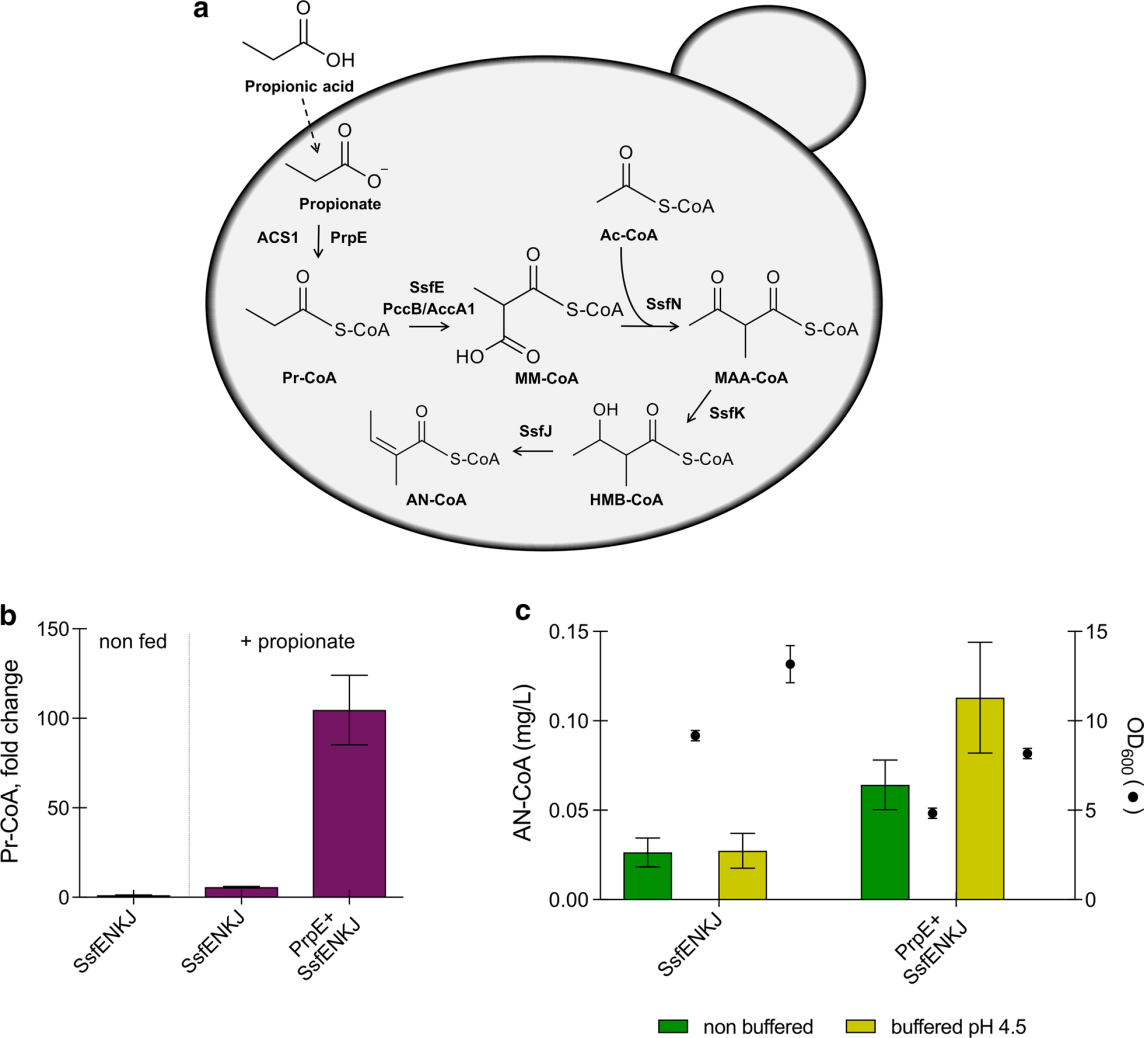
Intracellular accumulation of Pr-CoA and AN-CoA in *S. cerevisiae* strains expressing the *ssfENKJ* genes. **a** Graphical representation of the pathway starting with propionic acid feeding. **b** Pr-CoA accumulation in strains expressing *ssfENKJ*, and *ssfENKJ* together with *prpE*, shown as fold change compared to the respective control strains. Engineered strains were incubated in selective SC medium either non supplemented (“non fed”) or supplemented with 0.5 g/L propionic acid (“+ propionate”). **c** Intracellular accumulation of AN-CoA in strains ANG3 (*ssfENKJ*) and in strain ANG4 (*prpE *+ *ssfENKJ*) grown for 72 h in SC medium supplemented with 0.5 g/L propionic acid. The medium was buffered to pH 4.5 (yellow bars) or supplied unbuffered (green bars). Circles indicate OD_600_ at 72 h of growth. Represented are the averages and standard deviations of three independent cultures

Based on the hypotheses of Pickens et al. [6] we assembled the pathway to AN-CoA biosynthesis in baker’s yeast. First, we expressed the bacterial genes *ssfE*, *ssfN*, *ssfK,* and *ssfJ* as yeast codon-optimized versions for the conversion of Pr-CoA into AN-CoA (Fig. 2a). Pr-CoA is an intermediate metabolite produced in yeast through a variety of pathways including thio-esterification of propionate and catabolism of odd chain fatty acids and selected amino acids [15]. A plasmid, co-expressing the four heterologous genes under control of strong constitutive promoters was constructed together with a control plasmid (no ORFs downstream of the promoters). Transformation into yeast generated strains ANG1 (control) and ANG2 (*ssfE/ssfN/ssfK/ssfJ*). These strains were tested for production of AN-CoA. In strain ANG2 around 0.37 mg/L of AN-CoA accumulated, whereas no AN-CoA could be detected in strain ANG1 (control strain, data not shown).

**Fig. 3:**
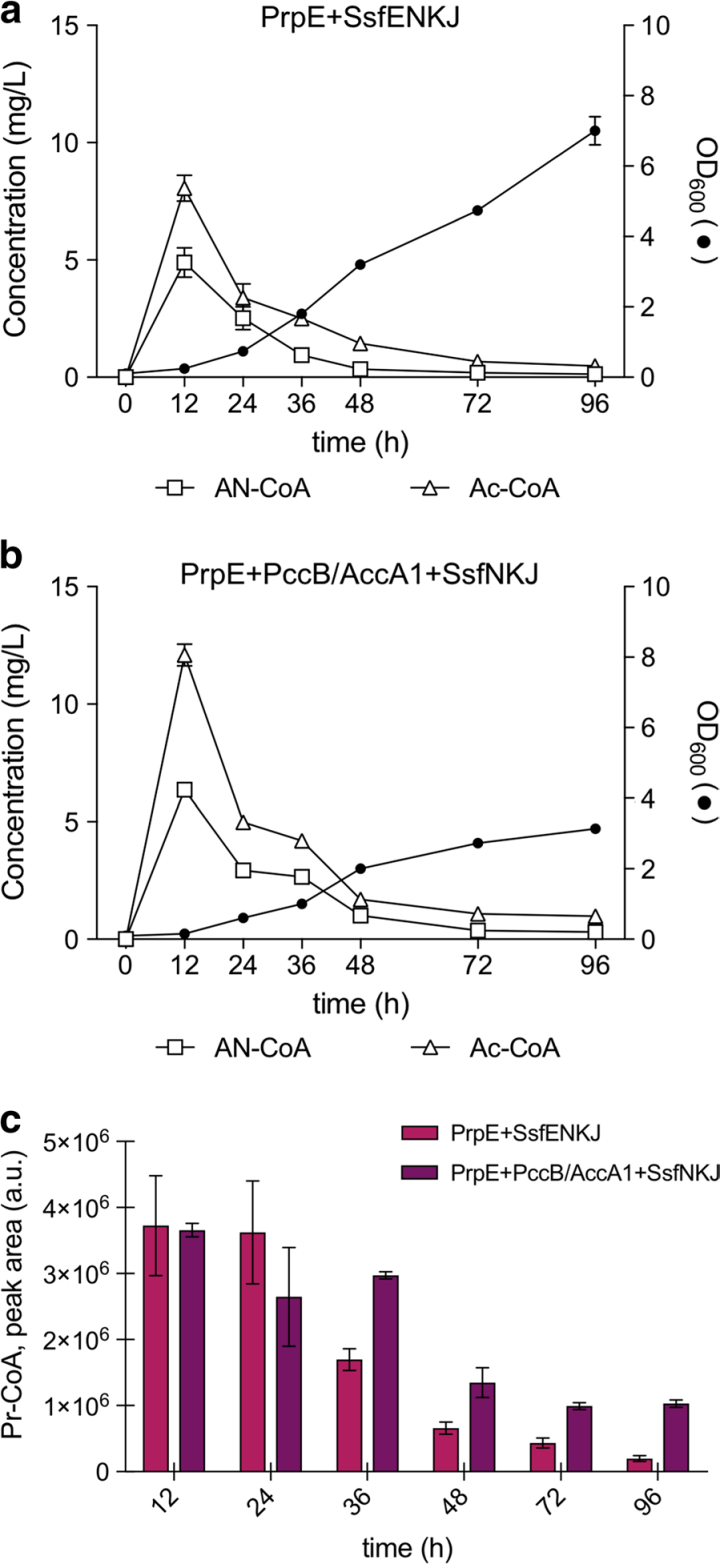
Time course of production of AN-CoA, Ac-CoA and Pr-CoA. **a** Ac-CoA and AN-CoA produced by strain expressing *prpE *+ *ssfENKJ* (ANG4). **b** Ac-CoA and AN-CoA produced by strain expressing *prpE *+ *pccB/accA1 *+ *ssfNKJ* (ANG5). **c** Relative amounts of Pr-CoA in strains expressing *prpE *+ *ssfENKJ* (ANG4) and strains expressing *prpE *+ *pccB/accA1 *+ *ssfNKJ* (ANG5). Strains were grown in SC medium supplemented with 0.5 g/L propionic acid and buffered to pH 4.5. Filled circles in **a**, **b** indicate OD_600_. Represented are the averages and standard deviations of three independent cultures

To test whether an improved precursor supply may increase AN-CoA synthesis, we cultured the strains in propionic acid-supplemented medium. Propionic acid is metabolized by the yeast via activation to Pr-CoA, which is catalyzed by ACS1—an endogenous isoenzyme of acetyl-CoA synthase [16]. ACS1 can accept propionic acid as substrate, albeit at lower rates than acetic acid [17]. To further improve propionate thio-esterification, we then expressed an acyl-CoA synthase specific for this anion. We chose to express propionyl-CoA synthase *prpE* from *Salmonella enterica* serovar Typhimurium, encoding an enzyme required for the catabolism of propionate in this bacterium [18]. Two strains were generated, one expressing the *ssf* genes and an empty plasmid (ANG3), the other one expressing the *ssf* genes together with *prpE* under control of the PGK1 promoter (ANG4). Feeding with propionic acid led to 5.5-fold elevated intracellular accumulation of Pr-CoA in strain ANG3, compared to the non-fed strain (Fig. 2b). Upon propionate supplementation, *prpE* expression in strain ANG4 boosted Pr-CoA accumulation to 20-fold higher values than those seen with strain ANG3 not expressing *prpE* (Fig. 2b). This is in accordance with an earlier report on *prpE* expression in the presence of exogenous propionate, leading to substantial accumulation of Pr-CoA in yeast [19]. The elevated concentration of Pr-CoA inside the cells resulted in a massive increase in AN-CoA production (from 0.37 mg/L to almost 5 mg/L at 12 h of growth, see Fig. 3a) but it also affected cell growth. The increased energy requirements needed to maintain cytosolic pH homeostasis in the presence of propionic acid in the medium may, in addition, contribute to growth retardation [20]. We cultured the cells in medium buffered to pH 4.5 as this pH value is a compromise between support of a decent yeast growth and remaining below the pK_a_ of propionic acid (pK_a_ = 4.88). This pH supports passive diffusion of propionic acid into the cells [21]. Cells cultured at pH 4.5 showed better growth and a slightly elevated production of AN-CoA (Fig. 2c).

We then performed time course experiments with strain ANG4 (*prpE *+ *ssfE/ssfN/ssfK/ssfJ*; Fig. 3a) and a newly constructed strain, ANG5 (*prpE *+ *pccB/accA1/ssfN/ssfK/ssfJ*; Fig. 3b). The latter strain expresses the propionyl-CoA carboxylase complex from *Streptomyces coelicolor* [22], instead of *ssfE*. The complex consists of the transcarboxylase subunit PccB and the biotin carrier protein/biotin carboxylase subunit AccA1 (PccB/AccA1 complex). Both strains were grown in buffered, propionate-supplemented medium. AN-CoA formation peaked at 12 h, but could barely be detected after 48 h of culture. AN-CoA accumulation reached maximally 4.9 ± 0.5 mg/L in strain ANG4 (Fig. 3a), whereas it climbed to even 6.4 ± 0.2 mg/L in strain ANG5 after 12 h of growth (Fig. 3b). Similar dynamics were detected for Ac-CoA, a critical metabolite for growth and proliferation. Ac-CoA accumulation also peaked at 12 h and went down to almost 0 upon 96 h of shake flask culture. In order to correlate AN-CoA production with Pr-CoA production, we also analyzed the relative amount of Pr-CoA accumulation in strains ANG4 and ANG5. Both strains accumulated similar levels of Pr-CoA within the first 12 h. Thereafter, in strains expressing *pccB/accA1*, Pr-CoA remained stable up to at least 36 h of growth. Even after 96 h a significantly higher level of Pr-CoA could be detected in ANG5 expressing *pccB/accA1* than in ANG4 expressing *ssfE* (Fig. 3c).

### Further analyses of the angelyl-CoA pathway in yeast

In order to investigate the individual functions of the *ssf* genes involved in AN-CoA biosynthesis in vivo, three truncated pathways were assembled on plasmids and transformed into yeast (strains ANG6-ANG8). The three strains expressed *prpE* together with one (*ssfE,* ANG6), two (*ssfE/ssfN,* ANG7), or three (*ssfE/ssfN/ssfK,* ANG8) genes from the *ssf* pathway. These strains were analysed together with the control strain expressing solely *prpE* (ANG9), and the strain expressing the entire pathway (*prpE *+ *ssfE/ssfN/ssfK*/*ssfJ,* ANG4). Strains were analysed for production of Pr-CoA, AN-CoA and the putative intermediates MM-CoA, MAA-CoA and HMB-CoA (see Additional file 1: Figure S2 for extracted ion chromatograms of all strains).

**Fig. 4:**
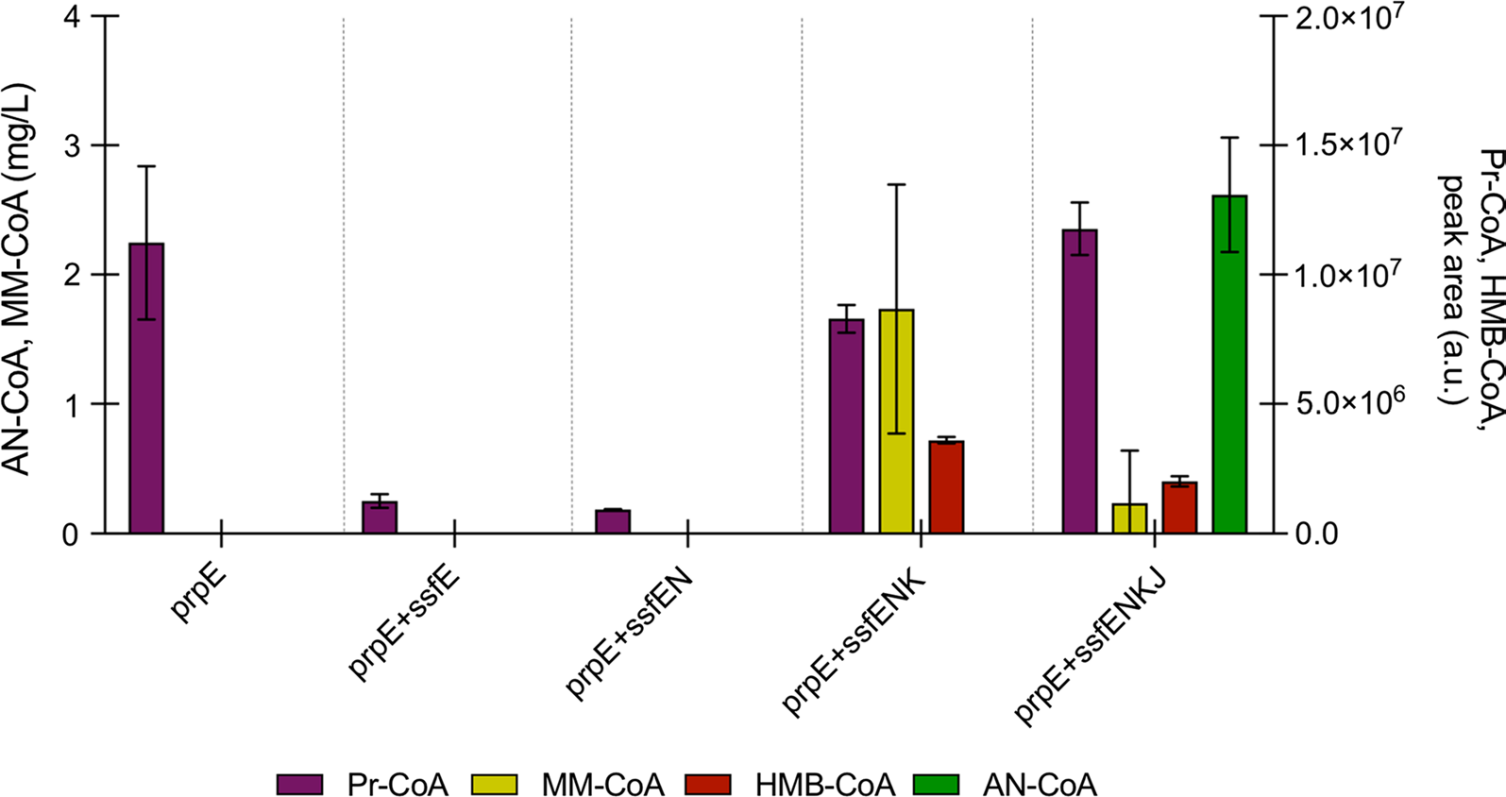
Intracellular accumulation of Pr-CoA, MM-CoA, HMB-CoA, AN-CoA in strains expressing parts of the AN-CoA pathway. MAA-CoA could not be found in any of the strains. Pr-CoA (purple bars), MM-CoA (yellow bars), HMB-CoA (red bars) and AN-CoA (green bars) in strains expressing *prpE* (ANG9), *prpE *+ *ssfE* (ANG6), *prpE *+ *ssfE/ssfN* (ANG7), *prpE *+ *ssfE/ssfN/ssfK* (ANG8) and *prpE *+ *ssfE/ssfN/ssfK/ssfJ* (ANG4). Engineered strains were incubated for 12 h in selective SC medium buffered to pH 4.5 and supplemented with 0.5 g/L propionic acid. Represented are the averages and standard deviations of three independent cultures

The strain expressing solely *prpE* (ANG9) accumulated only Pr-CoA (Fig. 4). The same is true for ANG6 (*prpE* and *ssfE* expressed). Neither MM-CoA nor MAA-CoA could be detected in strains expressing *prpE/ssfE/ssfN* (ANG7). However, accumulation of Pr-CoA in those strains was much lower compared to control strain ANG9, suggesting that Pr-CoA might have been utilized for further reactions inside the cells. When the pathway was extended to include *ssfK* (ANG8) yeasts produced HMB-CoA. In addition, a substantial accumulation of MM-CoA (1.7 mg/L) was detected in this strain, together with higher levels of Pr-CoA, compared to strains ANG6 or ANG7 (nearly seven- and ninefold more, respectively). Finally, strain ANG4, containing the entire pathway, accumulated AN-CoA together with lower amounts of HMB-CoA (nearly twofold less than ANG8) and MM-CoA (7.5-fold less than ANG11). Pr-CoA levels were comparable to those found in the control strain expressing only *prpE* (Fig. 4). Over time accumulation of HMB-CoA in strains expressing *prpE/ssfE/ssfN/ssfK* and accumulation of AN-CoA in strains expressing *prpE/ssfE/ssfN/ssfK/ssfJ* was accompanied by a decrease of the earlier intermediates (Additional file 1: Figure S3).

### Angelyl-CoA production in yeast starting from methyl-malonate

**Fig. 5:**
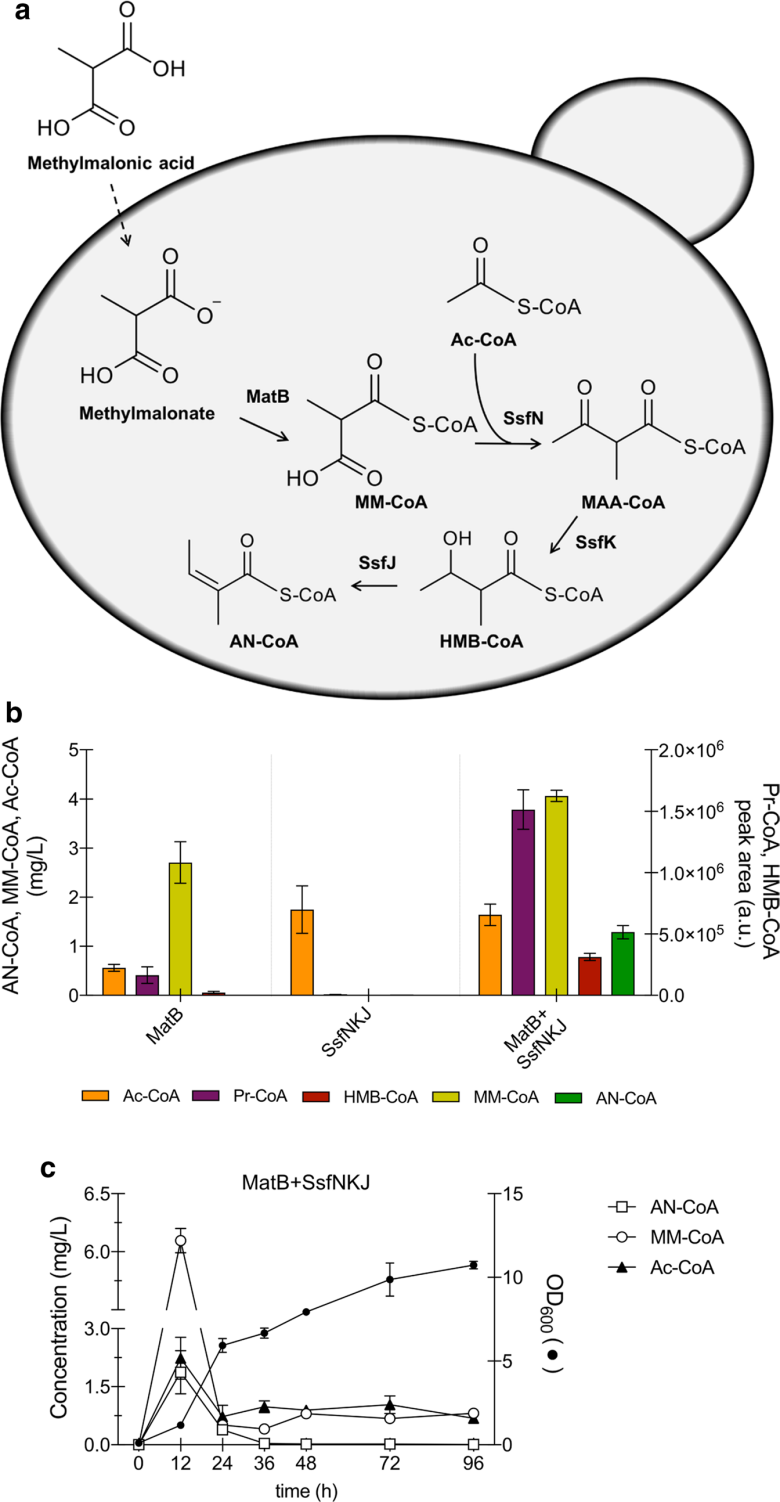
Impact of methyl-malonic acid feeding on AN-CoA production. **a** Graphical representation of the pathway starting with methyl-malonic acid feeding. **b** Intracellular accumulation of Ac-CoA (orange bars), Pr-CoA (purple bars), MM-CoA (yellow bars), HMB-CoA (red bars) and AN-CoA (green bars) in strains expressing *matB* (ANG10), *ssfNKJ* (ANG11) and *matB *+ *ssfNKJ* (ANG12), upon 12 h of growth. **c** Time course of production of AN-CoA (open squares), MM-CoA (open circles) and Ac-CoA (filled triangles) in strain expressing *matB *+ *ssfNKJ* (ANG12), and corresponding optical density at 600 nm (filled circles). All engineered strains were incubated in selective SC medium, supplemented with 0.5 g/L methyl-malonic acid (all data: mean ± SD, n = 3)

As an alternative route to AN-CoA production, we evaluated the malonyl/methylmalonyl-CoA ligase operating naturally in *Streptomyces coelicolor*. The biosynthetic route should allow for MM-CoA synthesis in yeast upon methyl-malonate supplementation and heterologous expression of malonyl-CoA synthase *matB* (Fig. 5a). MatB is an enzyme exhibiting a certain promiscuity, accepting both malonate and methyl-malonate as substrates [23]. Building of this pathway may avoid accumulation of potentially toxic amounts of Pr-CoA as it starts with a different substrate for production of methyl-malonyl-CoA.

Three plasmids were constructed and transformed into yeast, thus generating strain ANG10 (expressing *matB*), strain ANG11 (expressing *ssfN/ssfK/ssfJ*) and strain ANG12 (expressing the entire pathway *matB *+ *ssfN/ssfK/ssfJ*). Without feeding of methyl-malonic acid MM-CoA could not be detected in those strains (data not shown). Upon methyl-malonate feeding, expression of *matB* in strain ANG10 led to around 2.7 mg/L MM-CoA accumulation, representing the majority of the acyl CoA-pool (Fig. 5b). As expected, none of the compounds of interest could be detected in strain ANG7 expressing exclusively the *ssfN/ssfK/ssfJ* genes. Strain ANG12, expressing the entire pathway (*matB *+ *ssfN/ssfK/ssfJ*), was able to produce not only MM-CoA but also AN-CoA in titres in the range of 1.3–1.9 mg/L (Fig. 5b, c). Interestingly, also Pr-CoA substantially accumulated in strain ANG12 (*matB *+ *ssfN/ssfK/ssfJ*) (Fig. 5b). This was not observed in strain ANG10 or ANG11, suggesting that the accumulation was induced only when the full pathway was expressed. As shown with propionic acid feeding of strains ANG4 and ANG5 (Fig. 3a, b), AN-CoA accumulation peaked at 12 h of growth and declined thereafter to levels close to the limit of detection (Fig. 5c). MM-CoA accumulated in this strain to maximal titres of 6.1 ± 0.1 mg/L at 12 h.

### Angelic acid feeding and angelyl-CoA production

**Fig. 6:**
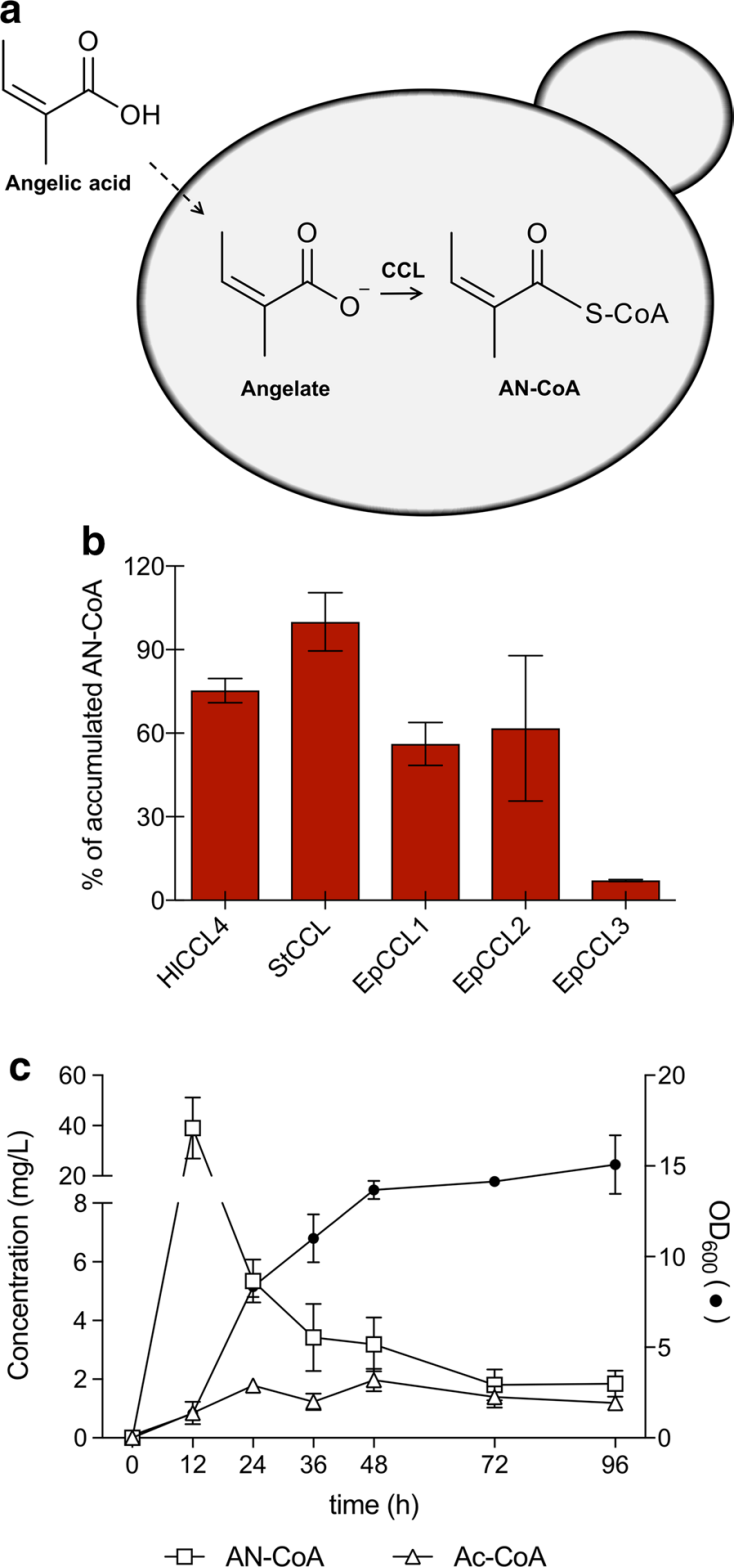
AN-CoA production in strains expressing plant acyl-CoA ligases and fed with angelic acid. **a** Angelate enters the cell and is converted to angelyl-CoA by carboxyl CoA ligases. **b** Relative amount of AN-CoA accumulating in yeast strains expressing CoA-ligases *HlCCL4* (ANG14), *StCCL* (ANG15), *EpCCL1* (ANG16), *EpCCL2* (ANG17), and *EpCCL3* (ANG18). Strains were cultured in selective SC medium supplemented with 0.1 g/L angelic acid. **c** Time course of production of AN-CoA (open squares) and Ac-CoA (open triangles) by the strain expressing CoA-ligase *StCCL* (ANG15). The corresponding OD_600_ values are represented by filled circles. Engineered strains were incubated in selective SC medium supplemented with 0.1 g/L angelic acid (all data: mean ± SD, n = 3)

In addition to setting up the entire pathway for angelyl-CoA production in yeast, we attempted to produce AN-CoA directly from angelic acid. We grew yeast strains expressing heterologous acyl-CoA ligases from plant, bacterial and fungal origin in angelic acid-supplemented medium (schematically shown in Fig. 6a). Acyl-CoA ligases can catalyze acyl-CoA thioester formation through adenylation of the carboxylic acid substrate. Many studies have explored the substrate specificity of these enzymes, revealing in several cases remarkable substrate promiscuities beyond their canonical substrate pools [24–26].

In preliminary experiments we found that two of the CoA-ligases tested showed the ability to accept angelic acid as a substrate for thio-esterification: carboxyl CoA ligase 4 from *Humulus lupulus* (HlCCL4) and predicted acyl-activating enzyme 6 from *Solanum tuberosum* (StCCL). The sequences of those two CoA ligases were used to search for CoA ligases in the available transcriptome of *Euphorbia peplus*, the plant producing ingenol-3-angelate. We identified several potential candidates. Three of them, arbitrarily named EpCCL1, EpCCL2 and EpCCL3, were prioritized based on their high sequence identity to HlCCL4 and StCCL (Additional file 1: Figure S4).

Three individual strains were constructed expressing the codon-optimized variants of *EpCCL1* (ANG16), *EpCCL2* (ANG17) or *EpCCL3* (ANG18). Strains ANG16, ANG17, and ANG18, in parallel with strains expressing *HlCCL4* (ANG14) and *StCCL* (ANG15), were assayed for AN-CoA production using medium supplemented with 0.1 g/L angelic acid. Figure 6b shows the relative activity of the CCL enzymes in yeast, expressed as percentage of AN-CoA accumulating in those cells. All of the putative CoA-ligases showed activity against angelic acid. Yeasts expressing the CoA ligase from *S. tuberosum* (ANG15) accumulated the highest amount of AN-CoA after 24 h. The amount of AN-CoA accumulating in those cells was set to 100%. ANG14, the strain expressing *HlCCL4*, accumulated 75% of this amount of AN-CoA, whereas yeasts expressing *EpCCL1* (ANG16) or *EpCCL2* (ANG17) accumulated 56 or 62% of this amount of AN-CoA, respectively. The strain expressing *EpCCL3* accumulated marginal amounts of AN-CoA (7% of the amount of ANG15). Expression of the heterologous enzymes had negligible effect on strain growth (data not shown).

Time course analysis of the *StCCL* expressing strain revealed a peak in AN-CoA production after 12 h, followed by a net decrease in titre (Fig. 6c). These kinetics were similar to the ones described before for AN-CoA production in yeast expressing the bacterial *ssf* pathway. The highest production level of AN-CoA at 12 h of batch culture was 39.0 ± 9.9 mg/L.

## Discussion

In this study we demonstrated the potential of yeast to produce AN-CoA by (A) heterologous expression of genes from a recently discovered gene cluster derived from actinomycetes, (B) the heterologous expression of plant acyl-CoA synthases able to accept angelic acid as substrate.

### Expression of the *ssf* pathway in *S. cerevisiae*

First, we showed production of AN-CoA in yeast through expression of part of the *ssf* pathway adopted from *Streptomyces* sp. SF2575. Pr-CoA is converted by SsfE to MM-CoA, which serves as substrate for the subsequent condensation reaction with Ac-CoA. Pr-CoA and Ac-CoA are both present in yeast, albeit the former only in small amounts. Upon propionate supplementation, ten times more AN-CoA (almost 5 mg/L) was produced in buffered medium when expressing an additional heterologous CoA-ligase, specific for Pr-CoA (propionyl-CoA synthase from *Salmonella*, PrpE).

Interestingly, the intracellular Pr-CoA concentration reached high levels, even when a second copy of the *ssfE* gene was expressed (Additional file 1: Figure S5). This doubling of the gene dosage should optimize coupling of Pr-CoA synthesis and the further downstream steps, pulling most of the Pr-CoA to AN-CoA. We propose that Pot1, the only 3-ketoacyl-CoA thiolase present in *S. cerevisiae*, may be responsible for Pr-CoA accumulation through the breakdown of the AN-CoA pathway intermediate MAA-CoA. Pot1 is normally involved in β-oxidation and it catalyses the conversion of 3-ketoacyl-CoA into an acyl-CoA shortened by two carbon atoms [27]. Shortening of MAA-CoA by two carbon atoms may inevitably lead to generation of Pr-CoA and Ac-CoA, thus explaining Pr-CoA build-up. The accumulation of Pr-CoA, besides being responsible for loss of carbon flux towards AN-CoA, may also be responsible for the growth inhibition suffered by those strains. Strains expressing *ssfE* reached OD_600_ values of ~ 7 after 96 h of growth, whereas strains expressing the *pccB/accA1* complex, instead of *ssfE*, underwent a more severe growth inhibition (OD_600_ = 3 after 96 h), parallel to the elevated accumulation of Pr-CoA (Fig. 3a, b). It has been described for both the filamentous fungus *Aspergillus nidulans* and the bacterium *Rhodobacter sphaeroides* that Pr-CoA inhibits enzymes involved in glucose metabolism, in particular CoA-dependent enzymes such as pyruvate dehydrogenase and succinyl-CoA synthase, leading to a significant growth retardation [28, 29]. In *Escherichia coli*, Pr-CoA was found to be a competitive inhibitor of citrate synthase [30]. Similar mechanisms could explain the severe growth retardation observed in yeast upon accumulation of Pr-CoA.

We attempted to overcome Pr-CoA toxicity by employing an alternative biosynthetic route that directly produced MM-CoA upon expression of methyl-malonyl-CoA synthase *matB* from *Streptomyces coelicolor*. This enzyme has recently been shown to generate methyl-malonyl-CoA in yeast upon methyl-malonate feeding [19]. Combining *matB* with the *ssf* pathway genes indeed induced generation of AN-CoA without severely hampering cell growth. Cells grown in those conditions could reach an OD_600_ of 10.7 after 96 h of growth, compared to OD_600_ of 3 and 7 when supplementing propionic acid (see Figs. 3, 5). Nevertheless, the levels of AN-CoA (1.5 mg/L) did not reach the values seen in yeast operating with the Pr-CoA pathway. The fact that expression of this alternative pathway also induces accumulation of Pr-CoA is consistent with the previously formulated hypothesis that Pr-CoA accumulation is induced via Pot1 activity on MAA-CoA.

The individual steps of the pathway were further characterized in vivo by sequential expression of the corresponding pathway genes. Contrary to expectations, no MM-CoA nor MAA-CoA could be detected in strains expressing either only *ssfE*, or *ssfE* together with *ssfN*. In cells expressing the entire pathway, accumulation of MM-CoA was observed—probably as a result of a “pulling effect” which led to substrate saturation of SsfN. MAA-CoA was never detected in any of the strains, even not in early phases of yeast growth, as confirmed by acyl-CoA analysis at 4 and 8 h (Additional file 1: Figure S3). MAA-CoA was also not detected in yeasts expressing genes from the *thg* cluster of *P. rubra* (Additional file 1: Figure S6), albeit the yeasts were able to produce AN-CoA. The activities of the enzymes ThgI, ThgK and ThgH, homologues to SsfN, SsfK and SsfJ, were characterized in vitro by Inahashi et al. [14], confirming that the AN-CoA pathway starts via MM-CoA and Ac-CoA condensation and runs through the intermediates MAA-CoA and HMB-CoA. Rapid conversion of MAA-CoA to either HMB-CoA or Pr-CoA and Ac-CoA (see before) might explain its analytical absence.

We have demonstrated production of AN-CoA up to 6.4 mg/L in yeast by introducing the bacterial *ssf* pathway. However, avenues remain to explore for further optimization of *S. cerevisiae*-based production of AN-CoA. Titres may e.g. be improved by insertion of higher copy numbers of rate limiting pathway enzymes, by promoter-based optimization of expression levels of individual enzymes, or by avoiding accumulation of pathway intermediates. Deletion of the non-essential gene *pot1* could also increase production levels. Compartmentalization of the heterologous pathway may also be a valuable approach for efficient production as it may increase spatial proximity of enzymes involved. To avoid feeding of propionic acid, insertion of a de novo production route to Pr-CoA is desirable. During submission of this manuscript, Krink-Koutsoubelis and colleagues reported a direct Pr-CoA production route from malonyl-CoA using parts of the 3-hydroxypropionate carbon assimilation cycle found in certain auxotrophic archaea and bacteria [31]. Coupling of this Pr-CoA biosynthesis pathway to the *ssf* pathway could enable AN-CoA production without addition of media supplements.

*Saccharomyces cerevisiae* has already been used for the biosynthesis of important precursors of esters of angelic acid, such as precursors of the diterpenoid ingenol-3-angelate [32]. Together with the reported AN-CoA synthesis, *S. cerevisiae* may provide an important and economic route to total biosynthesis of ingenol-3-angelate and other valuable angelates.

### Expression of acyl-CoA synthases from plant origin in *S. cerevisiae*

We also report the identification of plant acyl-CoA synthases able to utilize angelic acid in order to yield AN-CoA. In plants AN-CoA production most probably follows a different pathway than the one found in actinomycetes. AN-CoA may be derived from degradation of l-isoleucine (via 3-methyl-2-oxopentanoate and 2-methylbutanoyl-CoA) or from tiglyl-CoA through a *cis*–*trans* isomerase system, similar to that responsible for crotonyl-CoA isomerization [33, 34]. However, none of the responsible enzymes for its biosynthesis have been identified.

Here we attempted to enable ligation of angelic acid and CoA by employing heterologous enzymes known to have CoA ligation activity and substrate promiscuity. The enzyme HlCCL4 from *H. lupulus*, involved in bitter acids biosynthesis pathway in hop, was shown to have substrate preference towards several short-chain fatty acids, including isobutyric acid and 2-methylbutyric acid [35]. Obviously, the ability of HlCCL4 to utilize angelic acid as substrate can be attributed to the structural resemblance of the saturated acids that are the usual preferred substrates of the enzyme. The enzyme StCCL, showing 71% identity to HlCCL4 (Additional file 1: Figure S4), showed the highest activity and led to the highest titres of AN-CoA. Two of the enzymes identified in the transcriptome of *Euphorbia peplus* (EpCCL1 and EpCCL2) proved to be quite efficient in AN-CoA production, but not as much as StCCL. The *Euphorbia* CoA ligases are probably tightly linked to ingenol biosynthesis in the plant. Therefore, activity of those enzymes is synchronized with ingenol-3-angelate biosynthesis rather than being optimized for AN-CoA production. It may be also possible that AN-CoA synthesis does not go via the intermediate angelic acid in *Euphorbia peplus*.

Although angelic acid feeding might not be a relevant strategy for biotechnological production of AN-CoA, we envision that the strains expressing the CoA ligases identified in this work can provide a system for screening and functional characterization of acyl-transferases able to use angelyl-CoA as donor acyl-CoA. Such enzymes are needed for the transfer of the angelate moiety onto diverse acceptor molecules. More work will be necessary to identify these enzymes. We are currently screening the transcriptome of *Euphorbia peplus* for identification of possible candidates involved in ingenol-3-angelate biosynthesis. Candidates for this reaction are enzymes of the BAHD family of acyltransferases, as it has been recently shown for the esterification of hydroxycinnamoyl- and benzoyl-CoA [36]. Rapid acylation by BAHDs would probably also prevent the observed disappearance of angelyl-CoA, which may be due to intracellular hydrolysis of the activated compound, as previously observed for several CoA-activated molecules [37, 38].

## Conclusions

In this proof of concept study we have successfully achieved AN-CoA production in yeast by the expression of genes from the bacterial *ssf* cluster. This represents the first report on the activity of these enzymes in vivo. Moreover, we have identified acyl-CoA ligases from different plant species that use angelic acid as substrate and yield considerable titres of AN-CoA. Our results pave the way for future microbial production of different kinds of angelates.

## Methods

### Chemicals and media

All chemicals were bought from Sigma-Aldrich (St. Louis Missouri, USA) unless stated otherwise. Authentic standard of angelyl-CoA was synthesized by Jubilant LifeSciences, India.

LB medium for growth of *Escherichia coli* was supplied from Carl Roth GmbH + Co. KG (Karlsruhe, Germany), and was supplemented with 100 μg/L of ampicillin for amplification of plasmids.

Yeast extract peptone dextrose (YPD) medium with 20 g/L glucose was used for growth of wildtype strains prior to transformation. For pre- and main cultures of transformed strains we used synthetic complete (SC) drop-out medium (Formedium LTD, Hustanton, England), supplemented with 6.7 g/L yeast nitrogen base, 20 g/L glucose and all amino acids necessary for the corresponding auxotrophy. For propionyl-CoA carboxylase-expressing strains medium was supplemented with additional biotin (20 μg/L). Basic amounts of biotin are routinely added to baker’s yeast cultures as the co-factor is not produced by the laboratory strain S288C [39].

For preparation of pH 4.5-buffered medium a 1.0 M stock solution of citrate buffer (sodium citrate and citric acid) was prepared. Buffer stocks were filter-sterilized and used at the final concentration of 100 mM.

Organic acid supplemented media were prepared as solutions containing 0.5 g/L (6.7 mM) propionic acid, 0.5 g/L (4.23 mM) methylmalonic acid, or 0.1 g/L (1.0 mM) angelic acid, respectively. Stock solutions (1.0 M) of methyl-malonic acid and angelic acid were prepared in deionized water and ethanol respectively.

### Plasmids and strains

Table 1 lists all plasmids constructed in this work. All coding sequences were synthesized by GeneArt^®^ (Thermofisher Scientific, Zug, Switzerland) as yeast-codon optimized versions. Standard cloning was done using the restriction enzymes *Hin*dIII HF and *Sac*II, and T4 DNA ligase from New England Biolabs (Ipswich, Massachusetts, USA) according to standard protocols [40]. *E. coli* XL10 Gold (Agilent, Santa Clara, California, USA) cells were used for subcloning of genes. Coding sequences were cloned in single expression vectors (ARS/CEN), or in entry vectors for assembly of multigene expression plasmids in vivo by homologous recombination (HRTs), as described by Eichenberger et al. [41]. Briefly, genes were cloned into entry vectors, carrying different combinations of promoters and terminators (“expression cassettes”), flanked by 60 base pair homology sequences. Helper cassettes containing (a) the autonomously replicating sequence, (b) a centromere region, and (c) the auxotrophy marker are also flanked by 60 base pair homology sequences. Expression cassettes as well as helper cassettes were released by one-pot digestions using *Asc*I (New England Biolabs). The digested mixtures were transformed into yeast. In case of negative expression control, corresponding empty entry vectors were added to the digestion mix.

**Table 1:** List of genes used in this work and plasmids they were cloned into

Plasmid	Promoter and gene	Plasmid type	Organism	Accession number
pANG1	pPGK1-ssfN	Entry vector	*Streptomyces* sp. SF2575	ADE34503.1
pANG2	pTEF1-ssfK	Entry vector	*Streptomyces* sp. SF2575	ADE34504.1
pANG3	pPDC1-ssfJ	Entry vector	*Streptomyces* sp. SF2575	ADE34505.1
pANG4	pGPD1-ssfE	Entry vector	*Streptomyces* sp. SF2575	ADE34513.1
pANG5	pTEF1-thgK	Entry vector	*Polymorphospora rubra* K07–0510	BAU79605.1
pANG6	pPDC1-thgH	Entry vector	*Polymorphospora rubra* K07–0510	BAU79602.1
pANG7	pTEF2-thgI	Entry vector	*Polymorphospora rubra* K07–0510	BAU79603.1
pANG8	pGPD1-pccB	Entry vector	*Streptomyces coelicolor* A3(2)	NP_629079.1
pANG9	pPGK1-accA1	Entry vector	*Streptomyces coelicolor* A3(2)	NP_733754.1
pANG10	pPGK1-matB	ARS/CEN	*Streptomyces coelicolor*	CAB86109.1
pANG11	pPGK1-prpE	ARS/CEN	*Salmonella enterica* serovar Typhimurium	AAC44817.2
pANG12	pGPD1-ssfE	ARS/CEN	*Streptomyces* sp. SF2575	ADE34513.1
pANG13	pCYC1- prpE	ARS/CEN	*Salmonella enterica* serovar Typhimurium	AAC44817.2
pANG14	pTEF1-HlCCL4	ARS/CEN	*Humulus lupulus*	AGA17921.1
pANG15	pTEF1-StCCL	ARS/CEN	*Solanum tuberosum*	XP_006350454.1
pANG16	pTEF1-EpCCL1	ARS/CEN	*Euphorbia peplus*	–
pANG17	pTEF1-EpCCL2	ARS/CEN	*Euphorbia peplus*	–
pANG18	pTEF1-EpCCL3	ARS/CEN	*Euphorbia peplus*	–

*Saccharomyces cerevisiae* strains generated throughout this study are listed in Table 2. All constructed strains were derived from strain NCYC 3608 (NCYC, Norwich, United Kingdom), a derivative of S288C, modified in our labs to add auxotrophophic markers (HIS, LEU, URA), and repair the petite phenotype according to Dimitrov et al. [42]. All yeast strains were stored in 25% glycerol at − 80 °C.

**Table 2:** List of strains constructed in this work

Strains	Description
ANG1	Control strain with empty plasmid (URA3)
ANG2	Contains expression cassettes of *ssfE/ssfN/ssfK/ssfJ* (URA3)
ANG3	Contains expression cassettes of *ssfE/ssfN/ssfK/ssfJ* (URA3) and an empty plasmid (LEU2)
ANG4	Contains expression cassettes of *ssfE/ssfN/ssfK/ssfJ* (URA3) and *prpE* (LEU2)
ANG5	Contains expression cassettes of *pccB/accA1/ssfN/ssfK/ssfJ* (URA3) and *prpE* (LEU2)
ANG6	Contains expression cassettes of *ssfE* (URA3) and *prpE* (LEU2)
ANG7	Contains expression cassettes of *ssfE/ssfN* (URA3) and *prpE* (LEU2)
ANG8	Contains expression cassettes of *ssfE/ssfN/ssfK* and *prpE* (LEU2)
ANG9	Contains expression cassettes of *prpE* (LEU2) and an empty plasmid (URA3)
ANG10	Contains expression cassettes of *matB* (LEU2) and an empty plasmid (URA3)
ANG11	Contains expression cassettes of *ssfN/ssfK/ssfJ* (URA3) and an empty plasmid (LEU2)
ANG12	Contains expression cassettes of *ssfN/ssfK/ssfJ* (URA3) and *matB* (LEU2)
ANG13	Contains expression cassettes of *ssfE/thgK/thgH/thgI* (URA3) and *prpE* (LEU2)
ANG14	Contains expression cassette of CoA ligase from *Humulus lupulus* (LEU2)
ANG15	Contains expression cassette of CoA ligase from *Solanum tuberosum* (LEU2)
ANG16	Contains expression cassette of CoA ligase-1 from *Euphorbia peplus* (LEU2)
ANG17	Contains expression cassette of CoA ligase-2 from *Euphorbia peplus* (LEU2)
ANG18	Contains expression cassette of CoA ligase-3 from *Euphorbia peplus* (LEU2)
ANG19	Control strain with empty plasmid (LEU2)
ANG20	Contains expression cassettes of *ssfE/ssfN/ssfK/ssfJ* (URA3), *prpE* (LEU2) and an empty plasmid (HIS3)
ANG21	Contains expression cassettes of *ssfE/ssfN/ssfK/ssfJ* (URA3), *prpE* (LEU2) and an additional copy of *ssfE* (HIS3)

### Yeast transformation and growth

Yeast transformation was performed using the lithium acetate method [43]. Transformants were grown on agar plates prepared with selective SC drop-out medium. Pre-cultures were grown for 24 h at 30 °C on an orbital shaker (160 rpm). Optical density at 600 nm (OD_600_) of a 1:40 dilution was measured in an Ultrospec 10 table top spectrophotometer (GE Healthcare, Little Chalfont, United Kingdom). Main cultures for production of angelyl-CoA were inoculated in 25 mL of medium at a starting OD_600_ of 0.1, and grown at 30 °C (160 rpm) for 12–96 h. Media supplemented with organic acids were used exclusively for the growth of main cultures.

### Sample preparation

We harvested 100 OD-units (~ 1 × 10^9^ cells) by centrifugation at 4000 rpm for 5 min. Cell pellets were re-suspended in 1 mL of water and re-pelleted in 2 mL screw cap tubes. Extraction from pellets was performed as described previously [44]. Briefly, cell pellets were re-suspended in 500 μL of 75% ethanol and shaken (1500 rpm) for 3 min at 95 °C in a Thermo-Shaker TS-100 (Axonlab, Reichenbach an der Fils, Germany). Cell debris was removed by centrifugation (4000 rpm, 5 min) and the liquid phase was transferred to a 96 deepwell microplate. The ethanol extracts were evaporated for 5 h at 35 °C using a Genevac HT4 (SP Industries 935 Mearns RoadWarminster, PA18974). Dried pellets were re-solubilized in 100 μL of 50 mM ammonium acetate. Remaining debris was removed by centrifugation (5 min at 4000*g*), and supernatants were used for analyses.

### Acyl-CoAs analysis

Analytical LC–MS was carried out using a Waters Xevo G2 XS TOF mass detector (Milford, Massachusetts, USA). Separation of the compounds was achieved on a Waters Acquity UPLC^®^ HSS T3 C18 column (1.7 μm, 2.1 mm × 50 mm) kept at 50 °C. Mobile phases were composed of (A) 1% acetonitrile, 99% water, 5 mM ammonium acetate, and (B) 10% acetonitrile, 90% isopropanol, 5 mM ammonium acetate. An elution gradient from 99% A to 0% A within 2 min at a flow rate of 0.5 mL/min was used. The mass analyzer was equipped with an electrospray source and operated in negative mode. Capillary voltage was 1.0 kV; the source was kept at 150 °C and the desolvation temperature was 500 °C. Desolvation and cone gas flow were 1000 and 150 L/h, respectively. For each compound of interest we calculated peak areas on the extracted ion chromatograms of the respective [M−H]^−^ ions, using a mass window of 0.02 Da. Angelyl-CoA, acetyl-CoA and methyl-malonyl-CoA were quantified using a linear calibration curve with authentic standards ranging from 0.03125 to 4 mg/L for all compounds. For other compounds without standards, Area-under-the-curve (AUC) values were calculated for a relative quantity of the compound. Concentration and AUC values were normalized per 100 OD units.

## Additional file

**Additional file 1.** Additional figures and table.

## Abbreviations

KAS III: beta-ketoacyl-(acyl-carrier-protein) synthase III
ACP: acyl-carrier-protein
AN-CoA: angelyl-CoA
Ac-CoA: acetyl-CoA
MM-CoA: methyl-malonyl-CoA
MAA-CoA: methyl-acetoacetyl-CoA
HMB-CoA: 3-hydroxyl-2-methyl-butyryl-CoA
Pr-CoA: propionyl-CoA
CCL: carboxyl CoA ligase
HlCCL4: carboxyl CoA ligase 4 from *Humulus lupulus*
StCCL: acyl-activating enzyme 6 from *Solanum tuberosum*
EpCCL1: CoA-ligase 1 from *Euphorbia peplus*
EpCCL2: CoA-ligase 2 from *Euphorbia peplus*
EpCCL3: CoA-ligase 3 from *Euphorbia peplus*
BAHD: BEAT (benzoyl alcohol O-acetyltransferase), AHCT (anthocyanin *O*-hydroxycinnamoyl transferase), HCBT (anthranilate *N*-hydroxycinnamoyl/benzoyl transferase), and DAT (deacetyl vindoline 4-*O*-acetyltransferase)
