# Supplementary material for: Biosynthesis of angelyl-CoA in Saccharomyces cerevisiae
Source: Microb Cell Fact. 2018 May 12;17:72. doi: 10.1186/s12934-018-0925-8 (PMC5948907; doi:10.1186/s12934-018-0925-8)
Supplement: Supplementary file 1 — Additional file 1. Additional figures and table. [file 12934_2018_925_MOESM1_ESM.docx]

**Additional Material**

**Biosynthesis of angelyl-CoA in *Saccharomyces cerevisiae***

**Roberta Callari^1, 2^, David Fischer^1^, Harald Heider^1^, and Nora Weber^1^***

1 Evolva SA, Duggingerstrasse 23, 4153 Reinach, Switzerland

2 Department of Plant and Environmental Sciences, University of Copenhagen, DK-1871 Frederiksberg C, Denmark

*Corresponding author email: [noraw@evolva.com](mailto:noraw@evolva.com)

**Figure S1**

**Structure of bioactive angelates**. For each compound the angelic acid moiety is highlighted in blue.

**
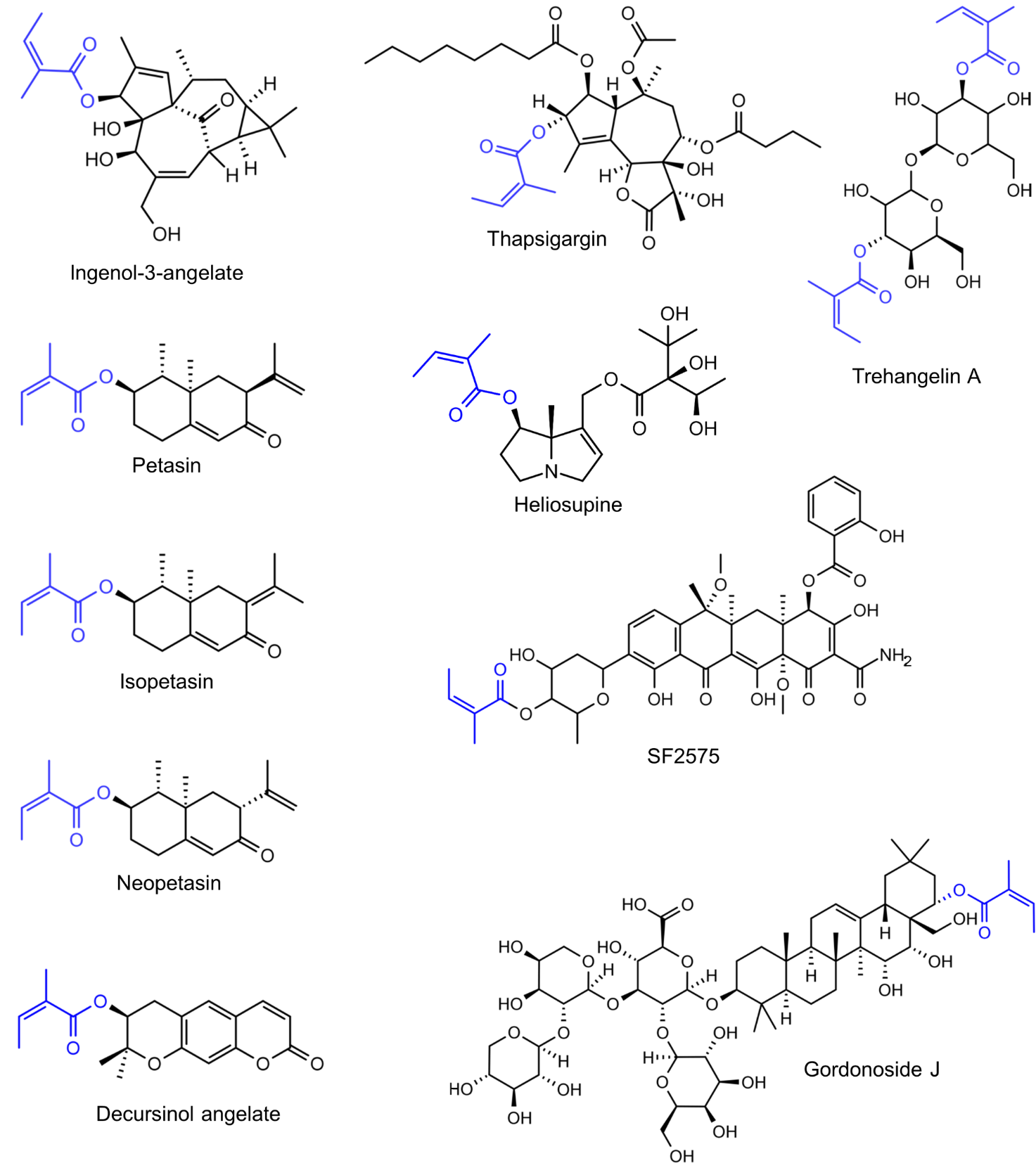
**

**Figure S2**


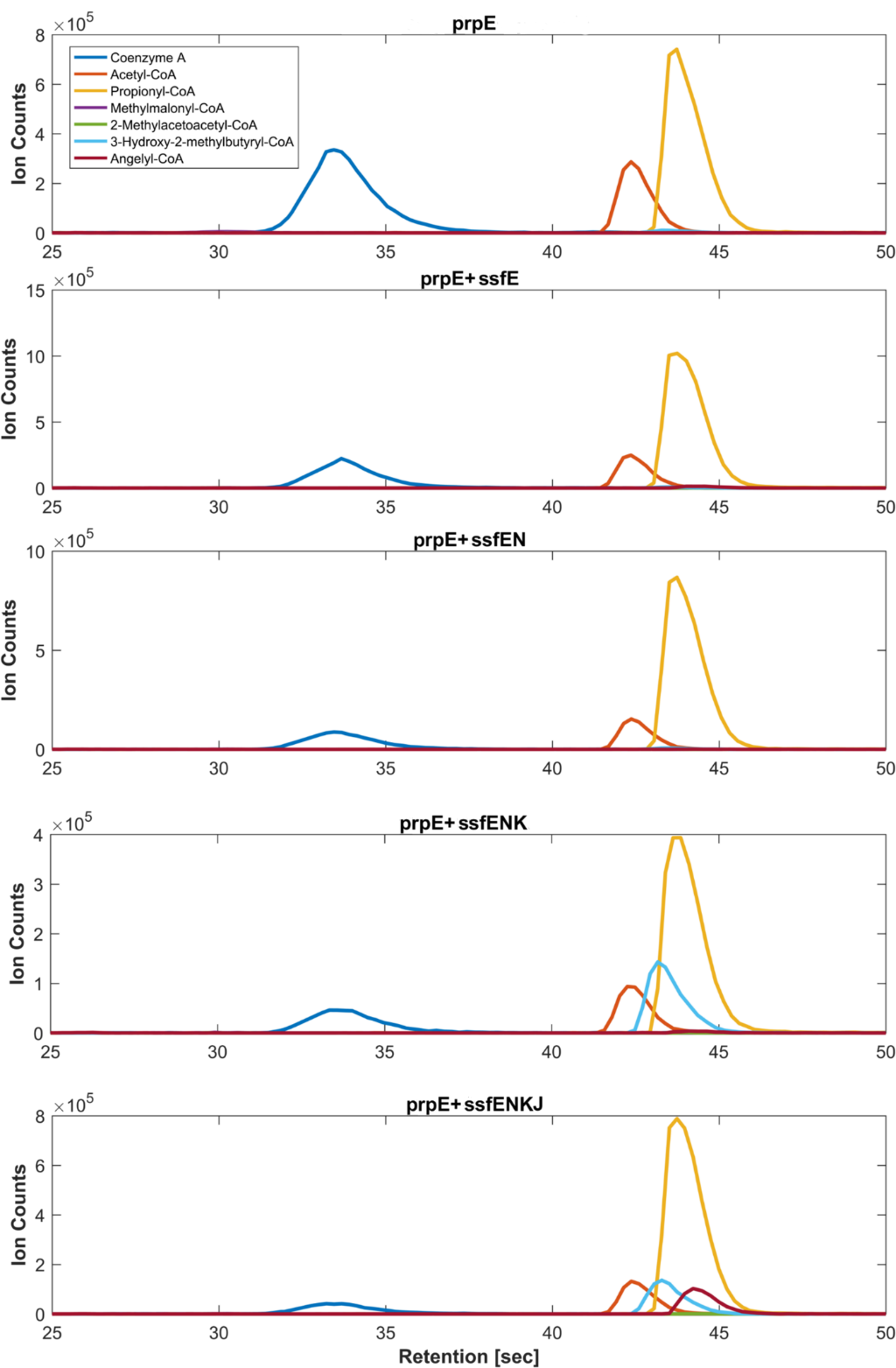
**Intracellular acyl-CoA analysis in strains expressing truncated or complete AN-CoA pathways.** Extracted Ion Chromatograms (EICs) of coenzyme A, Ac-CoA, Pr-CoA, MM-CoA, MAA-CoA, HMB-CoA and AN-CoA from strains expressing *prpE* (ANG9), *prpE+ssfE* (ANG6), *prpE+ssfEN* (ANG7), *prpE+ssfENK* (ANG8) and *prpE+ssfENKJ* (ANG4) grown for 12 h in selective SC medium buffered to pH 4.5 and supplemented with 0.5 g/L propionic acid.

**Figure S3**

**Intracellular acyl-CoA accumulation at 4, 8 and 12 h of growth.** Pr-CoA (purple bars), MM-CoA (yellow bars), HMB-CoA (red bars) and AN-CoA (green bars) in strains expressing **a.** *prpE*+*ssfE* (ANG6), **b.** *prpE*+*ssfE/ssfN* (ANG7) **c.** *prpE*+*ssfE/ssfN/ssfK* (ANG8) and **d.** *prpE*+*ssfE/ssfN/ssfK/ssfJ* (ANG4). Engineered strains were incubated for 4, 8 and 12 h in selective SC medium buffered to pH 4.5 and supplemented with 0.5 g/L propionic acid. Represented are the averages and standard deviations of three independent cultures.

**
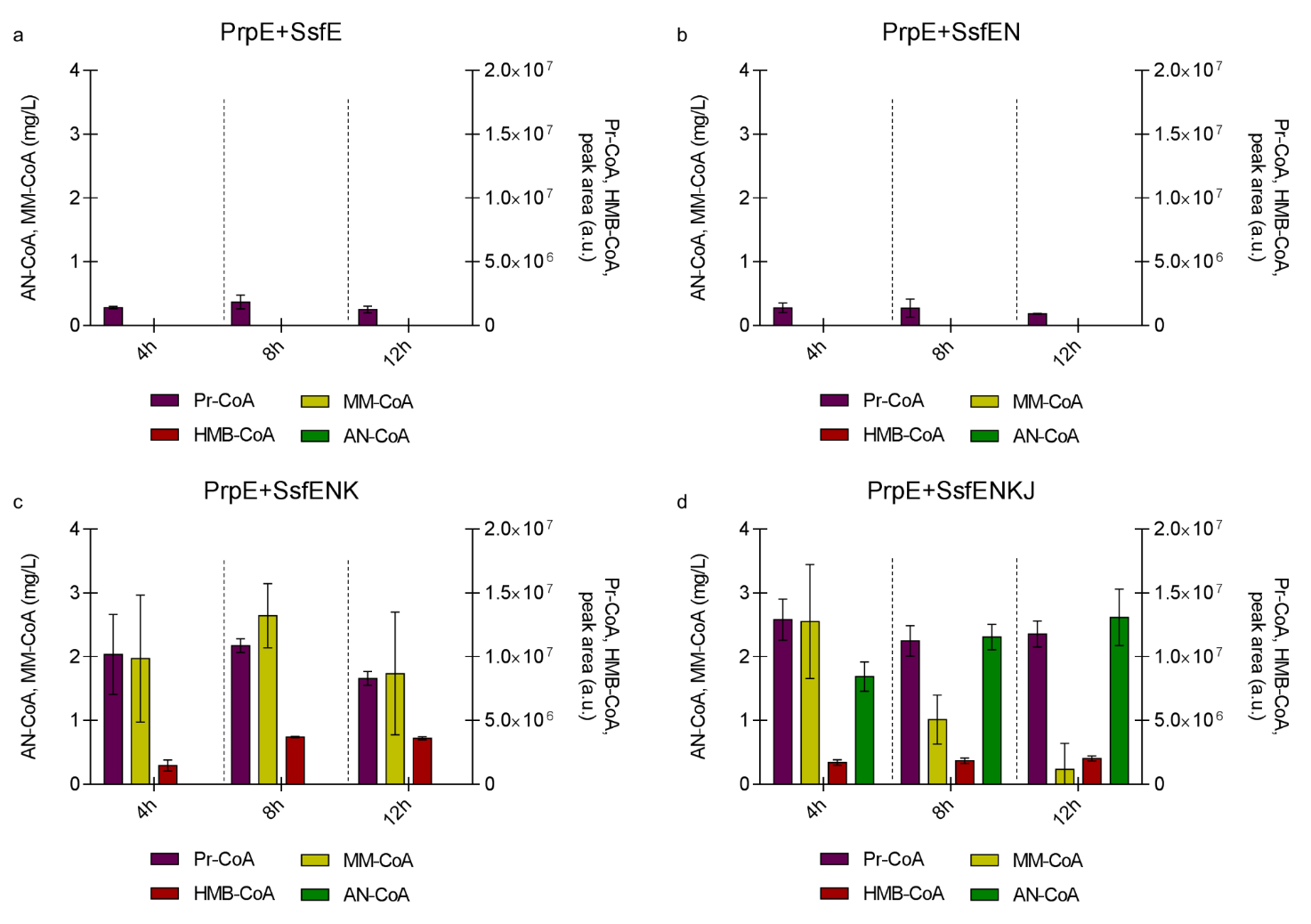
**

**Figure S4**

**Multiple protein sequence alignment of CoA ligases enzymes HlCCL4, StCCL, EpCCL1, EpCCL2 and EpCCL3 used in this study.** Sequences were aligned using T-Coffee (<http://tcoffee.crg.cat/apps/tcoffee/index.html>). Black shading indicates sequence identity.


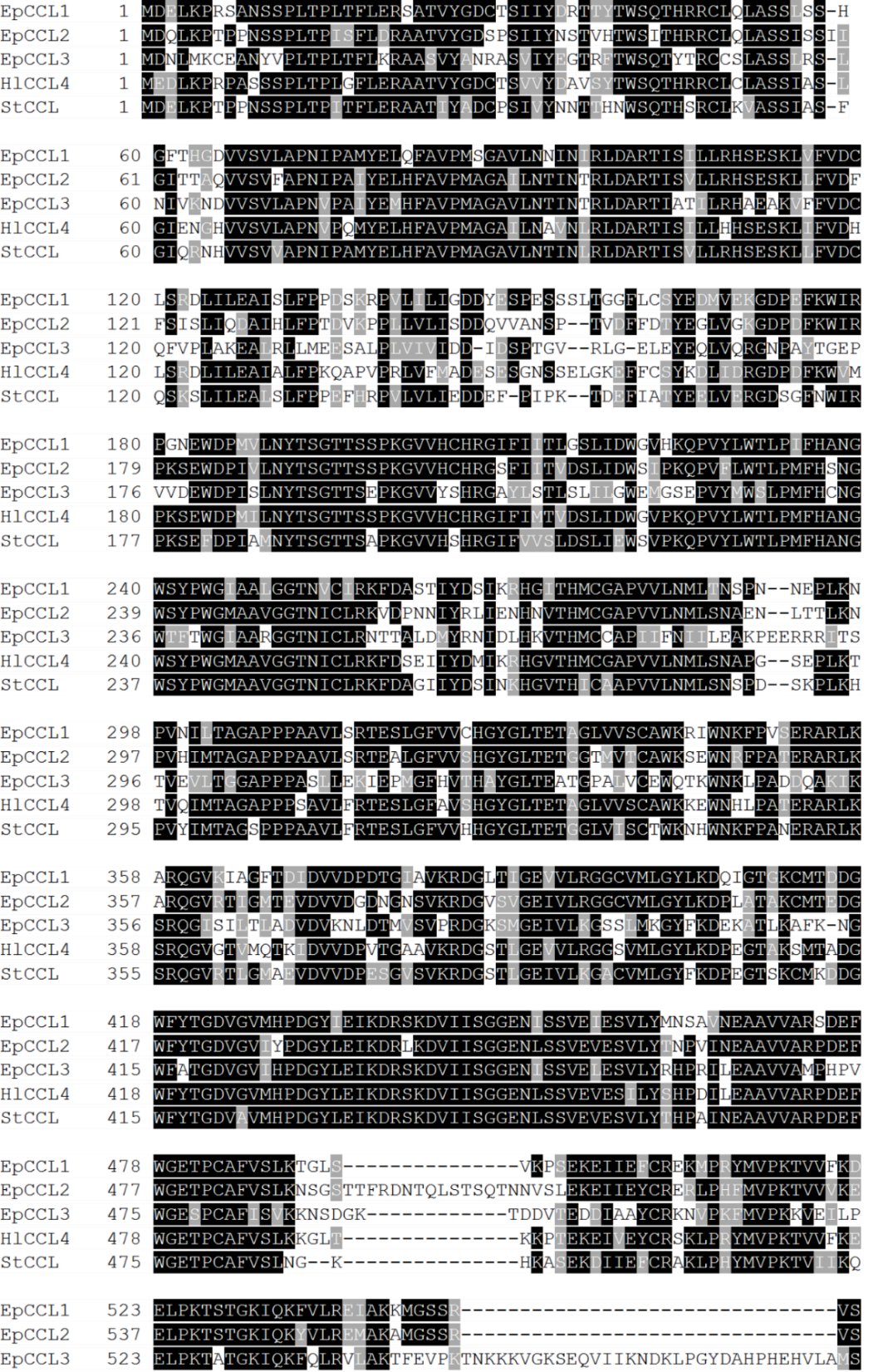


**FigureS5**

**Intracellular acyl-CoA accumulation in strains expressing one or two copies of *ssfE*.** Pr-CoA (purple bars), MM-CoA (yellow bars), HMB-CoA (red bars) and AN-CoA (green bars) in strains expressing *prpE+ssfE/ssfN/ssfK/ssfJ* (ANG20) and *prpE+ssfE/ssfN/ssfK/ssfJ* and an additional copy of *ssfE* (ANG21). Engineered strains were incubated for 12 h in selective SC medium buffered to pH 4.5 and supplemented with 0.5 g/L propionic acid. Represented are the averages and standard deviations of three independent cultures.


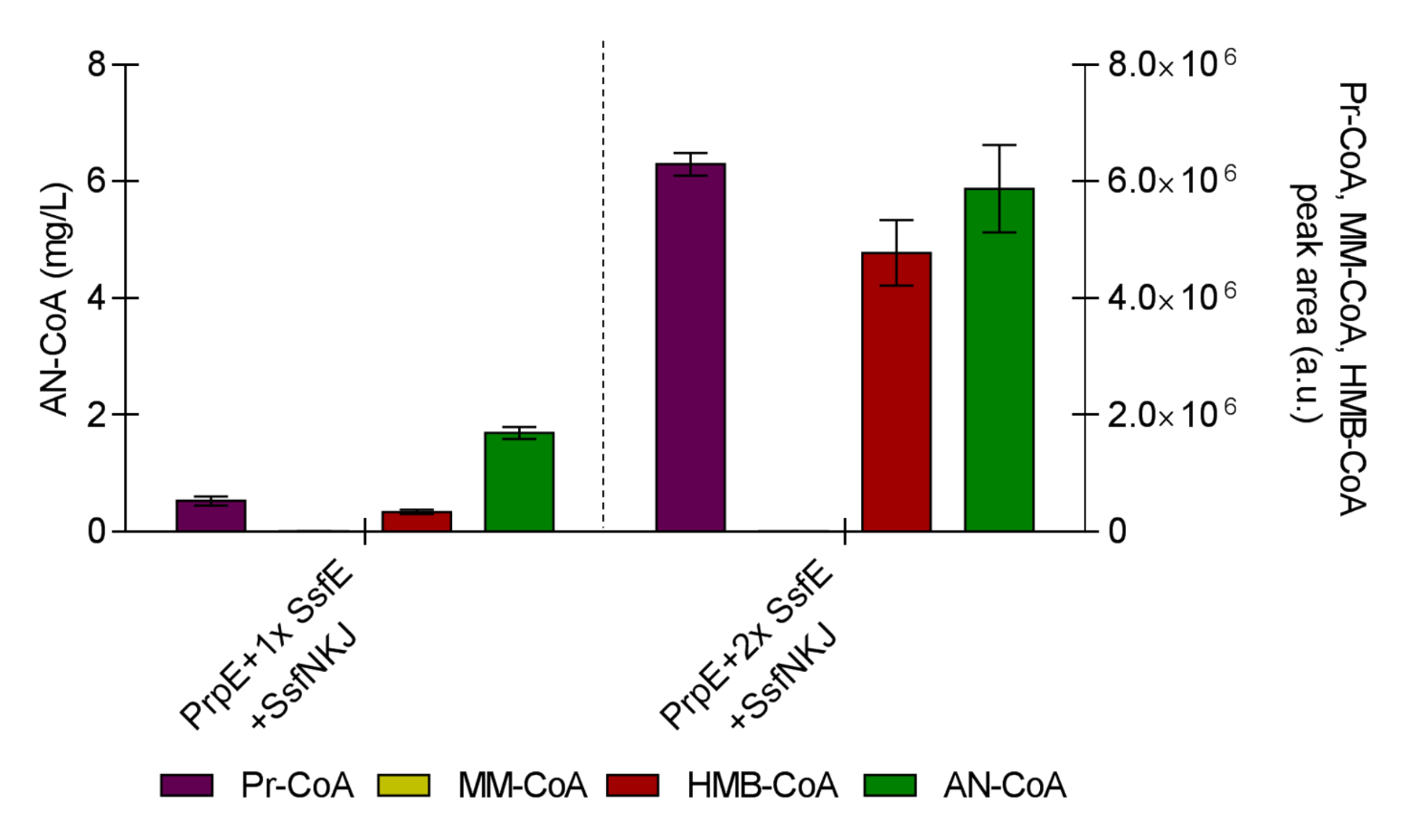


**Figure S6**

**Expression of the *thg* genes in yeast.** **a.** Graphical representation of the pathway starting with propionic acid feeding and including the *thg* genes. **b.** Intracellular accumulation of Pr-CoA (purple bars), MM-CoA (yellow bars), HMB-CoA (red bars) and AN-CoA (green bars) in strains expressing the propionyl-CoA synthase *prpE*, the propionyl-CoA carboxylase *ssfE* and the *thgIKH* genes (ANG13). Engineered strains were incubated for 12 h in selective SC medium buffered to pH 4.5 and supplemented with 0.5 g/L propionic acid (all data: mean ± SD, n = 3).


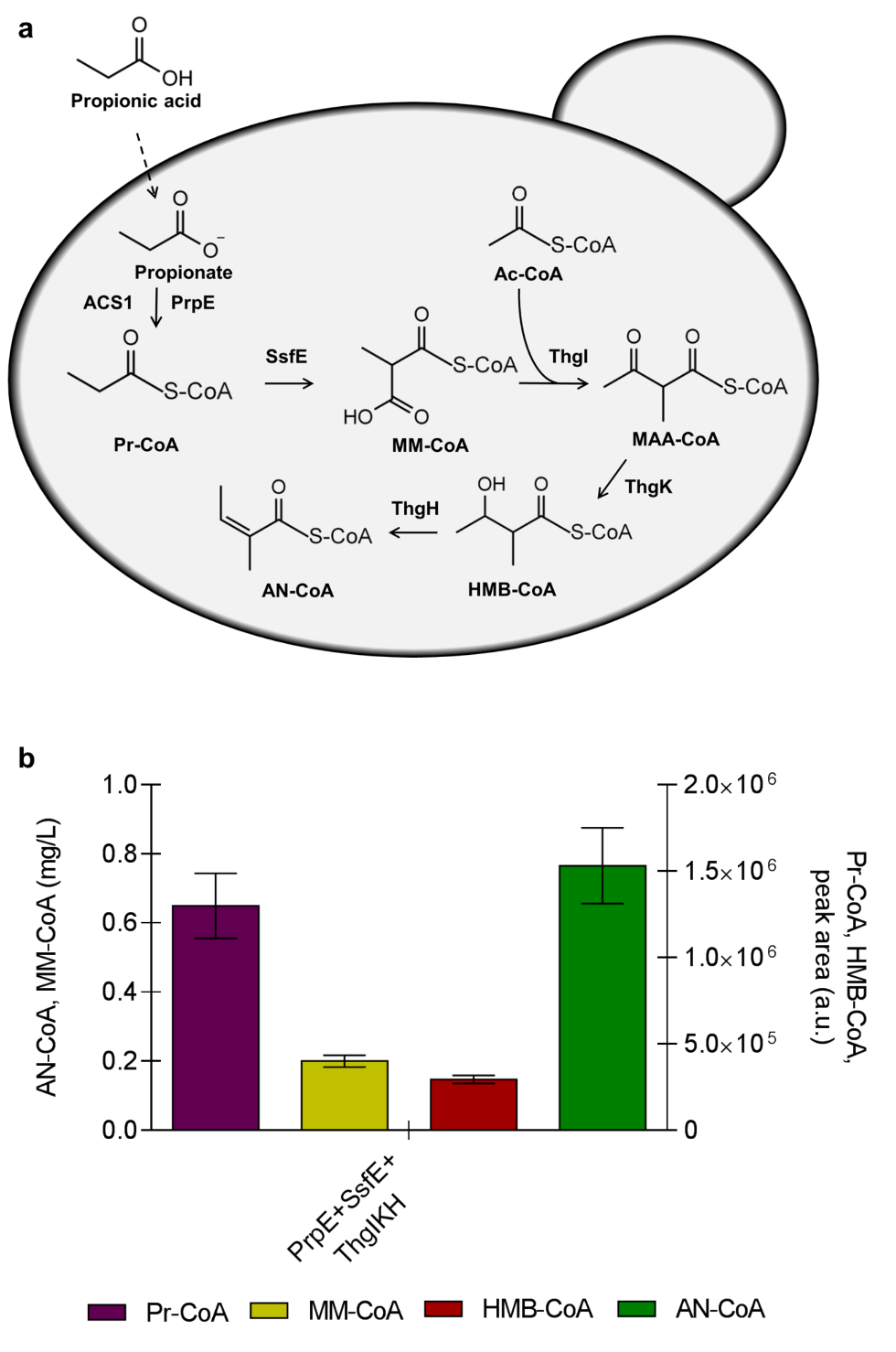


**Table S1.** **Coding sequences of genes used in this study.**

| **Gene** | **Yeast Codon optimized** | **Sequence** |
| --- | --- | --- |
| *ssfE* | yes | atggctgcctccagacatcattctgctactgctaaaccagctccagctggtccaagagaagctgaaagagctgttgaaggtgatgatgctcaaggtagagttgctgaattattggccattagagaacaagttagaagaggtccatctgaagctgctactcaagctcaaaaagctaagggtaaattgaccgccagagaaagaattgatttgttgttggacgaagattccttccacgaagttgaaccattgagaagacatagagctactggttttggtttggaagctagaagaccatataccgatggtgttattactggttggggtactgttcatggtagaactgtttttgtttacgcccacgatttcagaatttttggtggtgctttgggtgaagctcatgctacaaaaattcacaagattatggatatggctttggctgctggtgctccattggtttctttgaatgatggtgctggtgcaagaattcaagaaggtgtttctgctttagctggttacggtggtattttccaaagaaataccagagcttcaggtgtcattccacaaatctctgttatgttgggtccatgtgctggtggtgcagcttattctccagctttgactgattttgttttcatggtcagagaaacctcccaaatgtttattacaggtccagatgttgttcaagctgttaccggtgaacaaattactcaagatggtttgggtggtgccaacattcattctgttgttactggtgtttctcacttcgcttacgatgatgaacaatcttgcttggaagatgtcagatacttgttgtctttgttgccacaaaacaacagagaaactccaccagctgctgaatgtcatgatccagctgacagaagaggtgacgctttgttggatttggttccagctgatggtaatagagcttatgatatgcatgccgtcatcgaagaaatagttgatgacggtgaattcttagaaatccacgaacattggtccgccaacattatttgtgctttggctagattggatggtaaggttgttggtgttatagccaatcaacctaaatacttggctggtgtcttgaccattcaatcctctgaaaaagctgctagattcgttcaattgtgtgatgcctttaacatcccattggttaccttggttgatgttccaggttttttgccaggtgtttcacaagaacatggtggtattattagacatggtgccaagttgttgtacgcttactgtaatgctactgtcccaagaatttccgtcattttgagaaaggcatacggtggtgcatatatcgttatggattctcaatctattggtgccgatttgacttatgcttggccaactaacgaaattgctgttatgggtgctgaaggtgctgctaatgttattttcagaagacaaattgctgctgccgatgatccagatgctatgagagttagaatggtaaaagaatacaaggccgaattgatgcatccatattatgctgcagaaagaggtttggttgacgacgttattgatccagcagatacaagagcagttttgattgatggtttagccatgttgagaactaagcacgctgaattgccagctagaaaacatggtaatccaccacaatga |
| *ssfN* | yes | atggaaagaaccggtatcttgaacagaagatatgctggtccaggtgttactacttctgaattggctgctcatgctgttagagatttgttgtctagatctaccagaaccttggaagaagttggtttgactgttttggctacttctactccagatcaaccacaaccatctacagctgttagaatgcaaaatttgttgggtttgagatctaccccagcttttgatgttaatgctgtttgttctggtttcgtctacgctttgtctattgctgatgctatgttggctagagatggtggtactgctttggttgttggtgctgatatgtattcctctgttatggatagatccgacagaagaactgtttctttgtttggtgatggtgctgctgcaatgttgttgggtgaagttccagatggttatggtattcatgctacatctttattggccgatggtgactcttcagaattggttgaagttactgctggtggtacaagagaagctgctgatgaagcagctagagaagcaggtagacattatttcagaatgcaaggtagagctgtcagagattacgcattacattctatcccaaaggttatcggtgatgttttgggttctgctggtgtttcagctgatgatgttgatagattcatcgttcatcaaggtaacgctagattggtagaatctgttgctgatttgttaggtgttgaaatggctagagttccattgtctgctccattatatggtaatactggtgctgcagctttgccattgactttacatcattctcatagagaaagaccattggaaagaggtgaaagaattttgtttgctgctgttggtggtggtatgacagctggtgcagctttattgacttggtattga |
| *ssfK* | yes | atgagattgaaggataaggttgccgttattactggtggtactagaggtttgggtagagctattgctgaaagatatttggatgaaggtgcttctgttgtttgtgctgctagaaatccatacagaatcgatcaattggttgaacaattgccagacagagttatgtaccacgaaactgatgttacttccccagaatctgttgaaggtttgttggaagctgctgcaagaacttttggtagagttgatattttggttgctaacgccggtgttaatagagatggtaaggttgatagattggccgttgatgattggagagctatggttgataccaatttgtctggtacttacttctcattgagagctgctgccagattgatgactgctcaaggttctggtagaatcgttactgtttcttcatctatggcttccagagttgctgttggtgctggtggttattctgctacaaaagctgctattgaatgcttgactagagtttccgcaattgaattgggtccaaaaggtgttcaagttaactgtttggctccaggtgttttggatgatggtatgggtagagcattaaccgataatcataaggtttgggatgcttacagaaccagatttgccttaggtagagtcggtactttagatgaagctgctttgggtgctgtttttttggcttctgatgattcctcttacgttaacggtcatgttttggaagttaacggtggtttgttgtgggcttaa |
| *ssfJ* | yes | atgggtgtcaagtcccaagaaaccagagttagatttgaagtttctgaaggtgtcggtactatcttgttggatagagcaccagttaatgccttggatcatcaagctcaatctgaattgagagctgctgctgaagaagctgctcatagaactgatgttgctgctgttgtcttgtatggtggtcctgaagttttttctgctggtgctgatattagagaaatgggttctttgccagctgttgatatgagattgtgggctggtagattgcaaggtgcttttacagctgttgcagatattccaaaaccagttgttgctgcagttactggttatgctttgggtggtggttgtgaattggctttgactgctgatcatagaattttggctaccgaatccagaattggtttgccagaaatcaagttgggtgttattccaggtgctggtggtactcaaagattggctagattggttggtacttctagagccaagcaaatgatttttactggtagagctttgagagcagctgaagccttggaaattggtttagctgatcaagttgttccaagatccagagttttggctgaagctagagcttgggctagacaattcgttggtggtccagctttggctttaagagctgctaaacaagctattgatttgggtgctgcttctgatttgagaaccggtttagaaattgaaagaaccttgttcgaaggtttgttcggtactgaagatcaacaaactggtatgagatctttcgctgaaagaggtccaggtagagctgttttcacaggtagataa |
| *prpE* | yes | atgtccttcagcgaattctaccagaggtctattaacgaaccagaagccttttgggctgaacaagctagaagaattgattggagacaaccattcactcaaaccttggatcattctagaccaccatttgctagatggttttgtggtggtactaccaacttgtgtcataatgctgttgatagatggcgtgataagcaacctgaagccttggctttgattgctgtttcttctgaaactgacgaagaaagaactttcaccttctctcaattgcacgatgaagttaatatcgttgctgccatgttgttgtctttgggtgttcaaagaggtgatagagttttggtttacatgccaatgattgctgaagcccaaattactttgttggcttgtgctagaattggtgccattcattctgttgtttttggtggttttgcttcccattcagttgctgctagaatagatgatgctagaccagctttgatagtttctgctgatgctggtgctagaggtggtaagattttgccatacaaaaagttgttggatgacgctattgctcaagctcaacatcaacctaaacacgttttgttggttgatagaggtttagctaagatggcttgggttgatggtagagatttggattttgctaccttgagacagcaacatttgggtgcttcagttccagttgcttggttggaatctaacgaaacttcttgtatcttgtacacctcaggtactactggtaaacctaaaggtgttcaacgtgatgttggtggttacgctgttgctttggctacttctatggatacaattttcggtggtaaagctggtggtgtttttttctgtgcttctgatattggttgggttgttggtcattcttacatcgtttatgctccattattggctggtatggctactattgtctatgaaggtttgccaacttatccagattgtggtgtttggtggaaaatcgtcgaaaagtaccaagtgaacagaatgttttctgctccaaccgctataagagtcttgaaaaagtttccaaccgcacagatcagaaaccacgatttgtcatctttggaagccttgtatttggctggtgaaccattggatgaacctactgcttcttgggttactgaaactttaggtgttccagttattgacaactactggcaaactgaatctggttggccaattatggctttagctagagctttggatgatagaccatctagattgggttctccaggtgttcctatgtatggttacaacgttcagttgttgaacgaagttacaggtgaaccttgtggtatcaacgaaaaaggtatgttggttatcgaaggtccattgccaccaggttgtattcaaactatttggggtgatgatgccagattcgttaagacttattggtccttgttcaacagacaagtttacgctactttcgattggggtattagagatgctgaaggttactacttcattttgggtagaaccgatgatgtcattaacattgctggtcacagattgggtactagagaaatcgaagaatccatctcttcttacccaaacgttgcagaagttgctgttgttggtattaaggatgctttgaaaggtcaagttgccgttgcttttgttatcccaaaacaatctgataccttggctgatagagaagctgctagagatgaagaaaatgctattatggccttggttgacaatcaaatcggtcatttcggtagacctgctcatgtttggtttgtttcacaattgccaaagaccagatccggtaaaatgttgagaagaaccattcaagccatttgcgaaggtagagatccaggtgatttgaccactattgatgatccagcttccttgcaacaaattagacaagccattgaagagtaa |
| *pccB* | yes | atgtccgaaccagaagaacaacaaccagatattcataccactgctggtaaattggctgacttgagaagaagaattgaagaagctactcacgctggttctgctagagctgttgaaaaacaacatgctaagggtaaattgaccgccagagaaagaattgatttgttgttggatgaaggctccttcgttgaattggatgaatttgctagacacaggtctaccaattttggtttggatgctaatagaccatacggtgatggtgttgttactggttatggtactgttgatggtagaccagttgctgttttttcacaagatttcactgttttcggtggtgctttgggtgaagtttatggtcaaaaaatcgttaaggttatggacttcgctttgaaaactggttgtccagttgttggtattaacgattctggtggtgctagaattcaagaaggtgttgcttctttgggtgcttacggtgagatttttagaagaaatactcatgcctccggtgtcatcccacaaatttctttggttgttggtccatgtgccggtggtgcagtttattctccagctattactgatttcaccgtcatggttgatcaaacctctcatatgtttattaccggtccagatgttatcaagactgttacaggtgaagatgtcggtttcgaagaattaggtggtgcaagaactcataattctacatctggtgttgctcatcatatggctggtgacgaaaaagatgctgttgaatatgttaagcagctgttgtcttacctgccatctaacaatttgtctgaaccaccagcttttccagaagaagctgatttggctgttactgacgaagatgctgaattagatacaatcgttccagactctgctaatcaaccttacgatatgcattccgttatcgaacacgttttggatgatgccgaatttttcgaaactcaaccattattcgccccaaacattttgactggttttggtagagtagaaggtaggcctgttggtatagttgcaaatcaacctatgcaattcgctggttgtttggatattactgcttcagaaaaggctgccagattcgttagaacttgtgatgcttttaacgttccagtcttgactttcgttgatgttccaggttttttgccaggtgttgatcaagaacatgatggtattattagaaggggtgccaagttgatttttgcttatgctgaagctaccgttccattgattactgttattaccagaaaggcttttggtggtgcatatgatgttatgggttctaaacatttgggtgccgatttgaatttggcttggccaactgctcaaattgcagttatgggtgctcaaggtgctgttaatatcttgcatagaagaaccattgctgatgctggtgatgatgcagaagctacaagagctagattgatccaagaatatgaagatgccttgttgaacccatatactgctgctgaaaggggttatgttgatgctgttattatgccatctgataccagacgtcatatcgtcagaggtttaagacaattgagaacgaagagagaatctctgccaccaaaaaagcacggtaatattccattgtga |
| *accA1* | yes | atgaggaaggtcttgattgccaacagaggtgaaattgctgttagagttgctagagcttgtagagatgctggtattgcttctgttgctgtttatgctgatccagatagagatgcattacatgttagagctgctgatgaagcctttgctttaggtggtgatactccagctacttcctatttggatattgccaaagttttgaaggctgccagagaatctggtgctgatgctattcatccaggttatggttttttgtccgaaaacgctgaatttgctcaagctgttttggatgctggtttgatttggattggtccaccaccacatgctattagagatttgggtgataaggttgctgctagacatattgctcaaagagctggcgctccattggttgctggtactccagatccagtttctggtgcagatgaagttgttgcttttgctaaagaacatggtttgccaattgctattaaggctgcctttggaggtggtggtagaggtttgaaagttgcaagaactttggaagaggtcccagaattatacgattctgcagttagagaagctgttgctgctttcggtagaggtgaatgttttgttgaaagatacttggacaagccaagacacgttgaaactcaatgtttggctgatactcatggtaacgttgttgttgtttctaccagagattgctcattgcaaagaaggcatcaaaagttggttgaagaagctccagctccatttttgtctgaagctcaaactgaacagctgtactcttcatctaaggccattttgaaagaagctggttacgttggagctggtactgttgaatttttggttggtatggatggcaccatctcattcttggaagttaacactagattgcaagttgaacacccagttactgaagaagttgcaggtattgatttggtcagagaaatgttcagaattgccgatggtgaagaattgggttacgatgatccagctttgagaggtcattctttcgaattcagaatcaacggtgaagatccaggtagaggatttttgccagctccaggtactgttactttgtttgatgctccaactggtccaggtgttagattggatgccggtgttgaatctggttctgttattggtccagcttgggattctttgttggctaaattgatagttaccggtagaactagagctgaagccttgcaaagagcagctagagcattggatgaattcactgttgaaggtatggctactgctattccattccatagaactgttgttagagatccagcatttgctccagaattgactggttctactgatccttttactgttcataccagatggatcgaaaccgaatttgtcaatgagattaagccattcactaccccagctgatactgaaactgatgaagaaagtggtagagaaaccgttgttgtagaagttggtggtaagagattggaagtttctttgccatcttctttgggtatgtctttggctagaactggtttggctgctggtgctagaccaaaaagaagggctgctaaaaaatcaggtccagctgcttcaggtgatactttggcttctccaatgcaaggtactatcgttaagattgctgtagaagaaggtcaagaggttcaagaaggtgatttgatcgttgttttggaagccatgaagatggaacaaccattgaatgctcataggtccggtactattaagggtttgactgctgaagtaggtgcttctttgacttcaggtgctgctatttgtgaaatcaaggactga |
| *thgI* | yes | atgtccacctccaccgttattggtactggttcttatttgccaagaagggttttatcctctggtgaattggctagaagagttggtgttgctgaaaattggatcgttgaaaagaccggtatcagagaaagacgtgttgctgctgatgaagaagctacttctgatttggctactagagctgctagaagggctttgagaactgctagattggatccagctgatgttgatttgatcgttttggctacatctactccagatagaccaatgccagctactgcttctactgttcaagctaatttgggtgctagacaagctgttgctttcgatgttgatgctgtttgttctggttttgtttacgctttggttgttgcccattctatgttgaattctgaaggttgggctagaactgctttggttattggtgctgatacctactcaagagttttggattacactgatagaagaaccgctgttttgtttggtgatggtgctggtgcagttgttttgggtagagaaactggtggtggttctggtattagagctactactttgggtactgatggtactttggctgatttggttcaaattccagctggtggttcaagaaggccagcttctgctcaaactgttgaagctggtgatcattactttgctatgagaggtggtgatgttagaagattggctaatcaagttttcccagctttggtcggtcaattattgaaagctgcttctttggatttggatcaggttgacttgattgctgctcatcaagcaaatggtactatgttgactgattggtctagagatttgggtttaagaccaggtgttctgcatagaacagttgaaagatatggtaatactggtgctgcttctgttccagttactttggatgatgcagttagaacaggtagattgggtgctgctgctactttgttgatggttgcattcggtggtggtatgacttggggtggtgttgcattggattggtctgctgatccatctgttccaagatctaactgtgttaggtga |
| *thgK* | yes | atgactgctggtactggtcaaccagttgttgctgctccaggtagagctttggctgatagagttgttgttgttactggtgcttctagaggtgttggtagagacttggctagagtttttgctgatcatggtgctagattgggtttgttggctagatcaagagatgctttggatgacttaggtggtactttgactgctgctggtgctgatgttttggctgttccatgtgatgttggtgaaccagattctttggctggtgcagttgatgctgttgctggtcattttggtggtattgattctgttgttgtcaacgctggtatttctccagttgctagaagggctcatcatttgccaattgatgcttggcatgatgtcttggctactaatttgactggtggttttgttactgctagagctgcttatccacatttggcaagatctggtagaggtagattggttttcactacttctgttatggctgctactccaagaagaggtttgtctgcttatgctgcttctaaagctggtttggaaggtttgactagagctttagcagctgattgggctggtgatggtattttggttaatgcagttgctcctggttttttcgatactggtttgggtgctgcttttcatacttcacaaagattgcacgaacaagttgtaggtagaacaccagttgccagatttggtagagctgatgaattggctgctgcctttgtttttttggccggtgatgcttgtggttatttgacaggtcaagttttagctgttgatggtggttacggtttgggttaa |
| *thgH* | yes | atgtccgtttccagagttgttggttctgctgctactagaactgttactagaccagatgctccaggtccaccagttgctgctccagatccagctgatgctgttgttactgctgttgaaccatatgttgttagagccactattaacagaccagctagaagaaacgctattgatttggctgttatcgaaggtttggaaagagctattgacttagctgaagctactggtgctagagttttggttttgagaggtgctggtggtactttttgttctggtgctgatttgagagtcttggaagaaatgtctgttgatccacatagagtcgaaaccttcatggttagattggccttggttttaagaagattggaaaccgctagattcgtttctgttgcagttgttgaaggtcatgctgttgccggtggttgtgaaattttgttggcttgtgatgtttcagttgcagctacagatgctagaattggtgatagacatttggaatacggtttggttccagcagctggtggttctgttagattagctagaactttgccaaaagccagaggtaactatttgttgttggctgcagatttgttgactggtgaacaagctgctcaatggggtttagttagtgttgctgttccaccaactgatttggaacctagagttgatgctttggttggtagattggttggtcattctgctgatgctttagccgttgttaagaaaatggtttggactgctgatcatgaacctagacctgatgcaatgtcttgggaaagaagattattcttgagacacttgggttccgaagatgtttctgaaggtttgagagcttttagagaaagaagaaggccagctttcagagctgatgattaa |
| *matB* | yes | atgtcctctttgttcccagctttgtctccagctccaactggtgctccagctgatagaccagctttgagatttggtgaaagatcattgacttacgctgaattggctgctgcagctggtgctactgctggtagaattggtggtgcaggtagagttgctgtttgggctactccagctatggaaactggtgttgctgttgttgctgctttgttggctggtgtagctgctgttccattgaatccaaaatctggtgacaaagaattggcccacattttgtctgattctgctccatctttggttttggctccaccagatgctgaattgccaccagctttaggtgctttggaaagggttgatgttgatgttagagctagaggtgctgttcctgaagatggtgctgatgatggtgatccagctttggttgtttacacttctggtactactggtccaccaaaaggtgcagttattccaagaagggctttagctactactttggatgctttggctgatgcttggcaatggactggtgaagatgttttggttcaaggtttgcctttgttccatgttcacggtttggttttaggtattttgggtccattgagaagaggtggttctgttagacatttgggtagattttctactgaaggtgctgctagagaattgaatgatggtgcaactatgttgttcggtgttccaactatgtaccatagaattgctgaaactttgccagctgatccagaattggctaaagccttagctggtgcaagattattggtttctggttctgctgctttaccagttcatgatcacgaaagaattgcagctgctactggtagaagagttattgaaagatacggtatgaccgaaaccttgatgaatacttcagttagagctgatggtgaacctagagctggtacagttggtgttccattaccaggtgttgaattgagattggttgaagaagatggtactccaattgctgcattggatggtgaatctgttggtgaaattcaagttagaggtccaaacttgttcaccgaatacttgaatagaccagatgcaactgctgctgccttcaccgaagatggtttttttagaactggtgatatggccgttagagatccagatggttatgttagaatcgttggtagaaaggctaccgacttgattaagtctggtggttacaaaattggtgccggtgaaattgaaaatgccttgttggaacatccagaagttagagaagctgcagttactggtgaaccagatcctgacttgggtgaaagaatagttgcttggattgtcccagcagatccagctgctcctccagcattgggtactttagctgatcatgttgctgcaagattggctcctcacaaaagaccaagagttgttagatacttggatgccgttccaagaaatgatatgggtaagattatgaagagagccttgaacagagactaa |
| *StCCL* | no | atggatgagctaaagccaacgccaccaaattcaagtcctcttactcctattaccttcttggaaagagctgctactatctatgccgattgcccttccatcgtctacaacaacacaactcacaattggtcccaaacccattctcgttgcctaaaagttgcttcatccattgcatcttttggtattcaaagaaatcatgttgtctccgttgttgcccctaatatccctgccatgtatgagcttcattttgctgttcccatggctggtgctgtactcaacaccattaatctccgtcttgatgcacgtactatctctgtactcctccgtcacagcgaatctaaactcctcttcgttgattgtcaatccaaatccctaattctcgaagctctgtccttatttccgcctgaattccaccgtccggttctcgttcttatcgaggacgacgaattcccaattccaaaaactgatgaatttatcgctacttatgaggaattggttgaaagaggggattcgggtttcaattggattcgcccgaaaagtgaatttgatccgattgctatgaattacacttctggaactacatctgctccgaaaggtgtggttcatagccataggggtattttcgttgtttcgttggattcgttgattgaatggtccgttccgaaacagccggtttatttatggacgctacctatgtttcatgcaaacggatggagttatccatggggaatggctgctgttggtggaacgaatatctgtttgagaaaattcgatgccggaatcatttatgactcgatcaacaaacatggtgttactcatatctgcgctgctccagtggtactcaacatgttgtcgaattcccctgacagtaagccattaaaacaccctgtttatataatgacagcaggatccccaccccctgctgctgtcctgtttcgaacagagtcccttggatttgtagtccatcatggttatggacttacagaaactggtggattagttatttcttgtacatggaaaaatcactggaataaatttccagcaaatgaaagagcaaggctgaaatcaagacaaggggttaggacattagggatggcggaagtggacgtggtggatccagaatcaggagtcagtgttaaacgggacggatcaacattaggagaaattgttctaaagggtgcctgtgtcatgttgggttactttaaagacccggaaggaacgtcgaaatgcatgaaagatgatggttggttttacacaggggatgtggcagttatgcatcctgatggatacttagaaattaaagacagatcaaaggacgtgatcataagtggtggagagaatttgagcagtgtagaagtggaatcagtgttgtatacccatccagcgattaacgaagcagcagtagtggcacggccagatgaattctggggcgaaacaccgtgtgcatttgttagtctgaatggaaaacacaaggcgagtgaaaaagacattattgagttttgtagagccaaattgccacattatatggtaccaaagactgtcataattaaacaagagcttccaaagacatcaacagggaaaattcagaagttcgtgcttagagacattgctaaaagtatggggaaaagcaatagcagcaagaaggtgagcagaatgtag |
| *HlCCL4* | no | atggaagatctgaagccgagaccagccagctcctctccactcacccctctggggtttctggaaagagccgccaccgtttatggcgactgtacctccgtcgtttacgacgccgtttcatacacctggtcccagactcaccgccgctgtctctgtcttgcctcctccatcgcctcactcggcatcgaaaacggccatgtcgtctccgtcctcgccccaaacgtcccccaaatgtacgagcttcacttcgccgttcccatggccggcgccatcctcaacgccgtcaacctccgtctcgatgcccgcaccatctccatcctcctccatcacagcgaatcgaaactcatcttcgtcgatcatctctctcgtgatctcatcctcgaagccatcgctctgttcccgaaacaagcccctgttcctcgcctcgtttttatggcggacgagtctgaatcgggtaatagttcagagttggggaaagaattcttctgcagttataaggatctgatcgatagaggggacccggatttcaagtgggtcatgcctaaaagcgagtgggacccgatgattcttaactacacttctggaacgacgtcatcgccgaaaggggttgtccattgtcaccggggaatatttataatgacagtcgactctctcatcgattggggagttcctaaacagccagtttatctatggactctgcccatgtttcacgccaatgggtggagctatccttggggtatggcggcggtcggcgggaccaatatctgcctgcgtaaattcgactctgaaataatttacgatatgataaaacggcacggcgtgacccacatgtgcggagcccccgttgtactcaacatgctctccaacgcgccgggatcggaaccgctgaaaacaacggttcagatcatgactgcaggagctccgccgccctcggcggtgcttttccggaccgagtcgctgggcttcgcggtgagccacggctacgggcttaccgaaacggcggggttagtggtgtcgtgcgcgtggaagaaagagtggaaccatctcccggcgacggagagagcgaggctcaagtcgagacaaggggtggggacggtgatgcagaccaaaatcgatgtcgttgacccggtgaccggagccgccgtgaagcgagacggatcaacgttgggcgaggttgttctgagaggcgggtcggtcatgctcgggtacctaaaagacccagaaggaacggcgaaatccatgaccgcagacgggtggttctacaccggggacgttggagtcatgcacccagatgggtatttggagatcaaagaccggtccaaggacgtcatcatcagcggcggagagaatttgagcagcgtcgaggtggagtcaattctgtacagtcacccggatattctggaggcggcggttgtggcccggccagacgagttctggggggagacgccgtgtgctttcgtgagcttgaagaaaggtttaacgaagaagccgacggagaaggagatcgtggagtattgtcggagtaagttgccgcgttacatggtacccaaaacggtggtgtttaaggaggagcttcccaagacatcgactgggaaggttcagaaatttatactgagagatatggccagaggtatgggctctgcaactgctggagcgagccggagccgaatgtga |
| *EpCCL1* | yes | atggacgaattgaagccaagatccgctaattcttctccattgacaccattgactttcttggaaagatctgctactgtttacggtgattgcacctctattatctacgatagaactacttacacctggtcccaaactcatagaagatgtttacaattggcctcctccttatcttctcatggttttactcatggtgatgtcgtttctgttttggctccaaatattccagctatgtacgaattacaattcgccgttccaatgtctggtgctgttttgaacaacatcaacattagattggatgccagaaccatctccatcttgttgagacattctgaatccaagttggttttcgttgactgcttgtccagagacttgattttggaagctatttctttgttcccaccagattccaaaagaccagtcttgatattgatcggtgatgactatgaatccccagaatcttcttctttgactggtggttttttgtgctcctacgaagatatggttgaaaagggtgatccagaattcaagtggattagaccaggtaatgaatgggatccaatggttttgaactatacctctggtactacatcttctccaaagggtgttgttcattgccatagaggtattttcattatcaccttgggttccttgattgattggggtgttcataagcaaccagtttacttgtggactttgccaatttttcatgctaacggttggtcttatccatggggtattgctgctttgggtggtactaatgtctgtatcagaaagttcgatgcttccaccatctacgattccattaagagacatggtattacccatatgtgtggtgctccagttgttttgaatatgttgactaactccccaaacaacgaaccattgaagaacccagttaacattttgactgctggtgctcctccaccagctgctgttttatctagaactgaatctttgggtttcgtcgtttgtcatggttatggtttgactgaaactgctggtttggttgtttcttgtgcttggaaaagaatctggaacaagttcccagtttctgaaagagctagattgaaagctagacaaggtgttaagattgctggtttcaccgatatcgatgttgttgatccagatactggtattgccgtaaaaagagatggtttaaccattggtgaagttgtcttgagaggtggttgtgttatgttgggttacttgaaggatcaaatcggtactggtaagtgtatgactgatgatggttggttctatactggtgatgttggtgttatgcatccagatggttacatcgaaatcaaggatagatccaaggatgtcattatctctggtggtgaaaacatctcctccgttgaaattgaatctgtcttgtacatgaactccgctgttaatgaagctgcagttgttgctagatctgatgaattttggggtgaaactccatgtgctttcgtttctttgaaaactggtttgtccgttaagccatccgaaaaagaaattatcgaattctgcagagaaaagatgccaagatatatggttccaaagaccgttgttttcaaggatgaattgccaaagacttccaccggtaagattcaaaagttcgttttgagagaaatcgccaagaagatgggttcatctagagtttctagaatgtaa |
| *EpCCL2* | yes | atggaccaattgaagccaactccaccaaattcttctccattgactccaatctcattcttggatagagctgctactgtttatggtgattccccatctattatctacaactctactgttcatacctggtccattactcatagaagatgcttgcaattggcctcctccatttcttccattattggtattactaccgcccaagttgtttctgtttttgctccaaatattccagccatctacgaattgcattttgctgttccaatggctggtgctattttgaacactattaacaccagattggatgccagaaccatctctgttttgttgagacattctgaatctaagttgttgttcgtcgacttcttctctatctccttgattcaagatgccatccatttgtttccaaccgatgttaagccacctttgttggttttgatctccgatgatcaagttgttgctaactctccaactgttgatttcttcgatacttacgaaggtttggttggtaaaggtgatccagatttcaaatggatcagaccaaaatctgaatgggatccaatcgttttgaactatacttccggtactacatcttctccaaagggtgttgttcattgtcatagaggttccttcattatcaccgtcgattctttgattgattggtctattccaaagcaaccagttttcttgtggactttgccaatgtttcattctaacggttggtcttacccatggggtatggctgctgttggtggtactaatatttgtttgagaaaggtcgacccaaacaacatctacagattgatcgaaaaccataacgttacccatatgtgtggtgctccagttgttttgaatatgttgtctaacgctgaaaacttgaccaccttgaagaacccagttcatattatgactgctggtgctcctccaccagctgctgttttgtctagaactgaagccttgggttttgttgtttctcatggttacggtttgactgaaactggtggtacaatggttacttgtgcttggaaatcagaatggaatagattcccagctactgaaagagctagattgaaagctagacaaggtgttagaactatcggtatgactgaagttgatgttgttgatggtgataacggtaactccgttaagagagatggtgtttcagttggtgaaattgtcttgagaggtggttgtgttatgttgggttatttgaaagatccattggctaccgctaaatgcatgacagaagatggttggttttacactggtgatgttggtgttatctatccagacggttacttagaaatcaaggacagattgaaggacgtcattatttctggtggtgaaaatttgtcctccgtcgaagttgaatctgtcttgtacactaacccagttattaacgaagcagctgttgttgcaagaccagatgaattttggggtgaaactccatgtgctttcgtttctttgaagaactctggttctactaccttcagagacaacactcaattgtctacctctcaaactaacaatgtttccttggaaaaagaaatcatcgaatactgcagagaaagattgccacatttcatggttccaaagaccgttgttgtcaaagaagaattgccaaagacttccaccggtaagatccaaaagtatgttttgagagaaatggctaaggccatgggttcatctagagtttctagaatgtga |
| *EpCCL3* | yes | atggacaacttgatgaagtgcgaagccaattatgttccattgaccccattgacttttttgaagagagctgcttctgtttacgctaacagagcttcagttatctacgaaggtactagattcacttggtcccaaacttacactagatgttgttctttggcctcctcattgagatctttgaacatcgttaagaacgacgttgtttctgttttggctccaaatgttccagcaatctacgaaatgcactttgctgttccaatggctggtgctgttttgaacactattaacactagattggacgctagaaccattgccactattttgagacatgctgaagctaaggttttcttcgttgattgtcaattcgttccattggctaaagaagccttgagattattgatggaagaatccgctttgccattggttatcgttatcgatgatattgattctccaaccggtgttagattgggtgaattggaatatgaacaattggtccaaagaggtaacccagcttatactggtgaaccagttgttgatgaatgggatccaatttctttgaactatacctctggtactacctctgaacctaaaggtgttgtttattctcatagaggtgcttacttgtccaccttgtctttgattttgggttgggaaatgggttcagaaccagtttacatgtggtctttgccaatgtttcactgtaatggttggactttcacttggggtattgctgctagaggtggtactaatatttgtttgagaaacaccaccgccttggacatgtatagaaatattgacttgcataaggtcacccatatgtgttgcgctccaattatcttcaacattatcttggaagccaagccagaagaaagaagaagaattacttctaccgttgaagttttgactggtggtgctccaccaccagcatctttgttggaaaaaattgaacctatgggtttccatgttacccatgcttatggtttgactgaagctactggtccagctttggtttgtgaatggcaaacaaaatggaacaagttgccagctgatgatcaagccaagattaagtctagacaaggtatctccattttgaccttggctgatgttgatgttaagaacttggataccatggtttccgttccaagagatggtaaatctatgggtgaaatcgttttgaagggttcttcattgatgaagggttactttaaggacgaaaaggctactttgaaggcttttaagaatggttggtttgctactggtgatgttggtgtaattcatccagatggttacttggaaatcaaggacagatctaaggatgtcattatctctggtggtgaaaacatctcctccgtcgaattagaatctgtcttgtatagacacccaagaattttggaagctgctgttgttgctatgccacatccagtttggggtgaatctccatgtgcttttatctccgttaagaaaaactctgatggtaagaccgatgatgttaccgaagatgatatagctgcttactgcagaaagaacgttccaaagtttatggttccaaagaaggtcgaaatcttgccagaattgccaaaaactgctaccggtaagattcaaaagttccaattgagagttttggccaagactttcgaagttcctaagactaacaaaaaaaaggtcggtaagtccgaacaagtcatcatcaagaatgataagttgcctggttatgatgcccatccacatgaacatgttttagccatgtctagattgtga |
